# FGF12 Positively Regulates Keratinocyte Proliferation by Stabilizing MDM2 and Inhibiting p53 Activity in Psoriasis

**DOI:** 10.1002/advs.202400107

**Published:** 2024-09-05

**Authors:** Nan Wang, Xiejun Xu, Fangqian Guan, Yifan Lin, Yizhou Ye, Jie Zhou, Jianjun Feng, Sihang Li, Junbo Ye, Zhouhao Tang, Wenjie Gao, Bohao Sun, Yingjie Shen, Li Sun, Yonghuan Song, Litai Jin, Xiaokun Li, Weitao Cong, Zhongxin Zhu

**Affiliations:** ^1^ School of Pharmaceutical Science Wenzhou Medical University Wenzhou 325035 China; ^2^ Department of Pharmacy Zhejiang Provincial People's Hospital (Affiliated People's Hospital Hangzhou Medical College) Hangzhou 310014 China; ^3^ Department of Cardiology The Second Affiliated Hospital and Yuying Children's Hospital of Wenzhou Medical University Wenzhou 325027 China; ^4^ Department of Pathology The Second Affiliated Hospital of Zhejiang University Hangzhou 310009 China; ^5^ School of Life Sciences Huzhou University Huzhou 313000 China; ^6^ Department of Rheumatology and Immunology The First Affiliated Hospital of Wenzhou Medical University Wenzhou 325000 China; ^7^ Department of Orthopaedics The Second Affiliated Hospital and Yuying Children's Hospital of Wenzhou Medical University Wenzhou 325027 China

**Keywords:** FGF12, MDMD2, p53, proliferation, psoriasis

## Abstract

Psoriasis is a chronic skin disease characterized by abnormal proliferation and inflammation of epidermal keratinocytes. Fibroblast growth factor 12 (FGF12) is implicated in the regulation of diverse cellular signals; however, its precise mechanism in psoriasis requires further investigation. In this study, high expression of FGF12 is observed in the epidermis of skin lesion in psoriasis patients and imiquimod (IMQ)‐induced psoriasis like‐dermatitis. Moreover, specific loss of FGF12 in keratinocytes in IMQ‐induced psoriasis model alleviates psoriasis‐like symptoms and reduces proliferation. In vitro RNA sequencing demonstrates that knockdown of FGF12 effectively arrests the cell cycle, inhibits cell proliferation, and predominantly regulates the p53 signaling pathway. Mechanistically, FGF12 is selectively bound to the RING domain of MDM2, thus partially inhibiting the binding of β‐Trcp to MDM2. This interaction inhibits β‐Trcp‐induced‐K48 ubiquitination degradation of MDM2, thereby suppressing the activity of the p53 signaling pathway, which results in excessive cell proliferation. Last, the alleviatory effect of FGF12 deficiency on psoriasis progression is reversed by p53 knockdown. In summary, these findings provide valuable insights into the mechanisms by which FGF12 suppresses p53 signaling in keratinocytes, exacerbating the development of psoriasis. This positive regulatory loop highlights the potential of FGF12 as a therapeutic target to manage psoriasis.

## Graphical abstract

FGF12 is upregulated in the epidermis of patients with psoriasis and the imiquimod‐induced mouse model. Keratinocyte‐specific deletion of FGF12 ameliorates the psoriasiform phenotype. Mechanistically, FGF12 is selectively bound to the RING domain of MDM2, thus partially inhibiting the binding of β‐Trcp to MDM2. This interaction inhibits β‐Trcp‐induced‐K48 ubiquitination degradation of MDM2, thereby suppressing the activity of the p53 signaling pathway, which results in excessive cell proliferation.

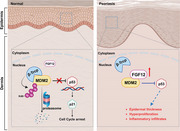

## 1 Introduction

Psoriasis is a common chronic inflammatory skin disease with characteristic skin lesions that are prone to recur and affects 2–3% of the global population.^[^ ^1^, ^2^ ^]^ Psoriasis has various clinical features, such as a thickened epidermis, excessive keratinization, abnormal blood vessel formation, and significant infiltration of immune cells in the skin.^[^ ^3^, ^4^ ^]^ Keratinocytes significantly impact the initiation and progression of psoriasis, playing a crucial role in the early onset and exacerbation of this disease.^[^ ^5^ ^]^ In response to a combination of genetic and environmental factors, keratinocytes are activated and can serve as a source of innate immune mediators.^[^ ^6^ ^]^ These include a range of pro‐inflammatory cytokines and chemokines, which are responsible for recruiting immune cells necessary for both innate and adaptive immune responses.^[^ ^7^ ^]^ Currently, cutting‐edge treatments for psoriasis involve the use of neutralizing antibodies targeting IL‐17, with clinical trials underway to test IL‐36 antagonists.^[^ ^8^ ^]^ Unfortunately, there are limitations, including high production costs, challenging administration methods, potential systemic side effects, and long‐term therapeutic resistance due to the development of drug resistance. Therefore, new and effective approaches to treat psoriasis must be developed.

The fibroblast growth factor (FGF) comprises ≈20 heparin‐binding proteins.^[^ ^9^, ^10^ ^]^ These proteins play crucial roles in various cellular processes involved in the development and repair of tissues, including the brain, skin, and lungs.^[^ ^11^, ^12^, ^13^ ^]^ FGF12, which consists of 243 amino acids, is an intracellular non‐secretory protein FGF. Its primary functional domain is the full‐length segment, including both the N‐terminal and C‐terminal disordered structures.^[^ ^14^ ^]^ FGF12 is highly expressed in the nervous system and plays a role in regulating voltage‐gated sodium channels in neurons.^[^ ^15^ ^]^ Additionally, FGF12 was reported to modulate nuclear factor‐κB and mitogen‐activated protein kinase signaling by interacting with specific proteins.^[^ ^16^ ^]^ FGF12 is an important regulator of vascular smooth muscle cell homeostasis in the circulatory system and thereby influences the progression of conditions such as atherosclerosis and pulmonary hypertension.^[^ ^17^ ^]^ Our recent study showed that loss of FGF12 in macrophages prevents liver fibrosis induced by bile duct ligation or carbon tetrachloride, suggesting that FGF12 inhibition in macrophages is a potential therapeutic approach for liver fibrosis.^[^ ^18^ ^]^ However, research of the role of FGF12 in psoriasis is limited, and it remains largely unknown whether FGF12 can regulate this disease. Further investigations are needed to explore the potential involvement of FGF12 in psoriasis and its clinical significance.

p53 is a well‐known tumor suppressor protein that plays a vital role in regulating various cellular processes and is primarily activated in response to cell stress and DNA damage.^[^ ^19^, ^20^ ^]^ When mild stresses or damage signals arise, p53 halts cell cycle progression, activates DNA repair mechanisms, and promotes cell survival.^[^ ^21^ ^]^ However, upon severe or widespread damage, p53 induces cell senescence or irreversible apoptosis, effectively eliminating cells with genomic instability and reducing the risk of cancer development.^[^ ^22^ ^]^ In addition to the DNA damage response, p53 is also involved in other cellular stress pathways.^[^ ^23^ ^]^ E.g., it can be activated in response to hunger or metabolic stress, leading to activation of the antioxidant response and autophagy,^[^ ^24^, ^25^ ^]^ which help to mitigate cellular damage caused by oxidative stress and maintain cellular homeostasis. However, the role of p53 in psoriasis has not been extensively investigated. Further research is needed to fully understand the involvement of p53 in the development and progression of psoriasis.

Mouse double minute 2 (MDM2) is an E3 ubiquitin‐ligase that is widely recognized as a significant negative regulator of p53.^[^ ^26^, ^27^ ^]^ MDM2 negatively regulates p53 by binding to it and promoting its ubiquitination, resulting in degradation of p53 by the 26S proteasome.^[^ ^28^, ^29^ ^]^ Additionally, MDM2 regulates p53‐mediated gene transcription by binding to p53 and inhibiting its activity.^[^ ^30^, ^31^ ^]^ The relationship between p53 and MDM2 is complex because p53 can stimulate transcription of MDM2.^[^ ^32^, ^33^ ^]^ Moreover, the protein stability of MDM2 is regulated by various factors, with two common mechanisms being 14‐3‐3‐σ binding‐mediated auto‐ubiquitination and degradation,^[^ ^34^ ^]^ as well as β‐Trcp‐induced degradation.^[^ ^35^ ^]^ Importantly, MDM2 is amplified and overexpressed in several human malignancies, including sarcoma, breast cancer, and brain tumors. The MDM2‐p53 signaling pathway in keratinocytes has been reported to promote cell proliferation by inhibiting p53 activity, thereby contributing to the development of squamous cell carcinoma of the skin.^[^ ^36^ ^]^ However, the role of MDM2/p53 in the progression of psoriasis and the underlying mechanism remains unclear.

The cocktail of IL‐17A, IL‐22, oncostatin M, IL‐1α, and TNF‐α (M5) cytokines induces keratinocytes manifesting some features of psoriatic keratinocyte in vitro, which has been applied to establish a psoriatic keratinocyte model in vitro.^[^ ^37^, ^38^ ^]^ Meanwhile, imiquimod (IMQ), an agonist of Toll‐like receptor 7/8 ligand, induced dermatitis in mice closely resembling human psoriasis.^[^ ^39^ ^]^ Therefore, we used M5 and IMQ to induce psoriatic models in vitro and in vivo, respectively. In the present study, we observed increased expression of FGF12 in psoriatic epidermal tissue. Furthermore, depletion or knockout of FGF12 reduced the abnormal proliferation capacity of keratinocytes and alleviated psoriasis symptoms, mainly through negative regulation of p53 signaling pathway. Furthermore, the inhibition of p53 activity by FGF12 depends on MDM2 in keratinocytes. Our mechanistic investigations revealed that FGF12 is bound to RING domain of MDM2, and subsequently stabilized MDM2 by inhibiting its β‐Trcp‐induced K48‐linked ubiquitination. This stabilization of MDM2 led to transcriptional inhibition of p53. In summary, this study uncovers a novel mechanism by which FGF12 promotes the development of psoriasis by regulating the activity of p53.

## 2 Results

### 2.1 FGF12 Is Upregulated in the Epidermis of Patients with Psoriasis and Imiquimod (IMQ)‐Induced Mouse Model

**Figure 1:**
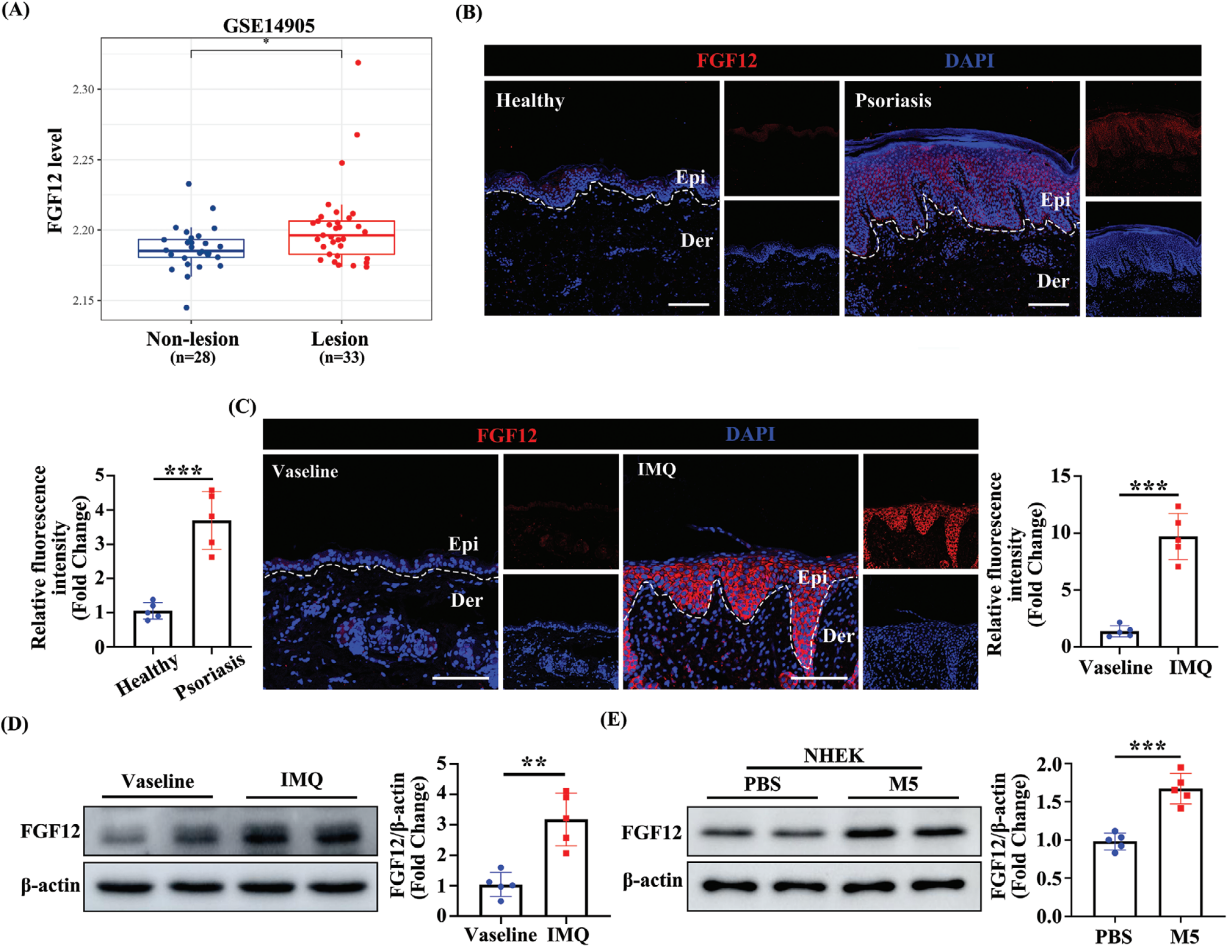
FGF12 is upregulated in the epidermis of patients with psoriasis and IMQ‐induced mouse model. A) The mRNA level of *Fgf12* in the skin from non‐lesion skin and lesion skin of psoriasis patients was analyzed using the Gene Expression Omnibus (GEO) database. B) Immunofluorescent and quantitative analysis of FGF12 in the skin from healthy controls and psoriatic patients. Nuclei were stained with DAPI (blue) (n = 5). Scale bar = 200 µm. C) Immunofluorescent and quantitative analysis of FGF12 in the skin from Vaseline and IMQ‐mice. Nuclei were stained with DAPI (blue) (n = 5). Scale bar = 100 µm. D) Immunoblotting and quantitative analysis of FGF12 expression in the skin from Vaseline and IMQ‐mice. β‐Actin was used as a loading control (n = 5). E) Immunoblotting and quantitative analysis of FGF12 protein level in NHEK cells that were treated with PBS or M5 for 12 h. β‐Actin was used as a loading control (n = 5). Error bars show the mean ± SEM. *p < 0.05; ***p < 0.001. The *p* value was determined using two‐tailed unpaired Student's t test (A – E). All numbers (n) are biologically independent experiments.

To investigate the association between psoriasis and FGF12, we initially analyzed the expression pattern of FGF12 in skin biopsies. Immunofluorescence staining showed that the FGF12 positive signal (red) predominantly co‐localized with the Krt14 positive signal (green) in the epidermis of both human psoriasis skin and the IMQ‐induced mouse model (Figure S1A,B, Supporting Information). This colocalization suggested that FGF12 was predominantly expressed in keratinocytes. Analysis of the Gene Expression Omnibus dataset GSE14905 revealed that *Fgf12* mRNA expression was significantly higher in lesional skin than in non‐lesion skin from psoriasis individuals (**Figure** **1**A). Immunofluorescence staining showed that FGF12 expression was markedly higher in epidermal keratinocytes of lesional skin from psoriasis patients than in normal skin from healthy donors, indicating that there is a potential correlation between FGF12 and the development of psoriasis (Figure 1B).

In addition, immunofluorescence staining showed that the level of FGF12 was elevated in the epidermis of IMQ‐induced psoriasis mice and was strongly correlated with the pathological progression of psoriasis (Figure 1C). Consistently, western blot analysis revealed that FGF12 expression was significantly higher in IMQ‐induced psoriasis mice than in normal control mice (Figure 1D). Moreover, we conducted corresponding verification with primary keratinocytes (NHEK) treated by M5,^[^ ^40^ ^]^ a pro‐inflammatory cytokines combination that induces psoriasis in vitro (Figure 1E). Immunofluorescence staining indicated that M5 treatment upregulated FGF12 expression in HaCaT cells (Figure S1C, Supporting Information). Similarly, western blot results revealed that the psoriasis model phenotype in vitro, including Akt and p65 pathways, were also activated after 12 h M5 treatment, along with FGF12 and cell proliferation factor Cyclin D1 (Figure S1D, Supporting Information). The qRT‐PCR results showed that after 12 h treatment, *Fgf1*2 levels peaked, along with the cell proliferation factor *Ccnd1*, and the inflammatory factors *IL‐1β* and *IL‐6* (Figure S1E, Supporting Information). Taken together, these data demonstrate that expression of FGF12 is upregulated in lesions and correlates with the exacerbation of psoriasis.

### 2.2 FGF12 Ablation in Keratinocytes Ameliorates the Psoriasiform Phenotype

**Figure 2:**
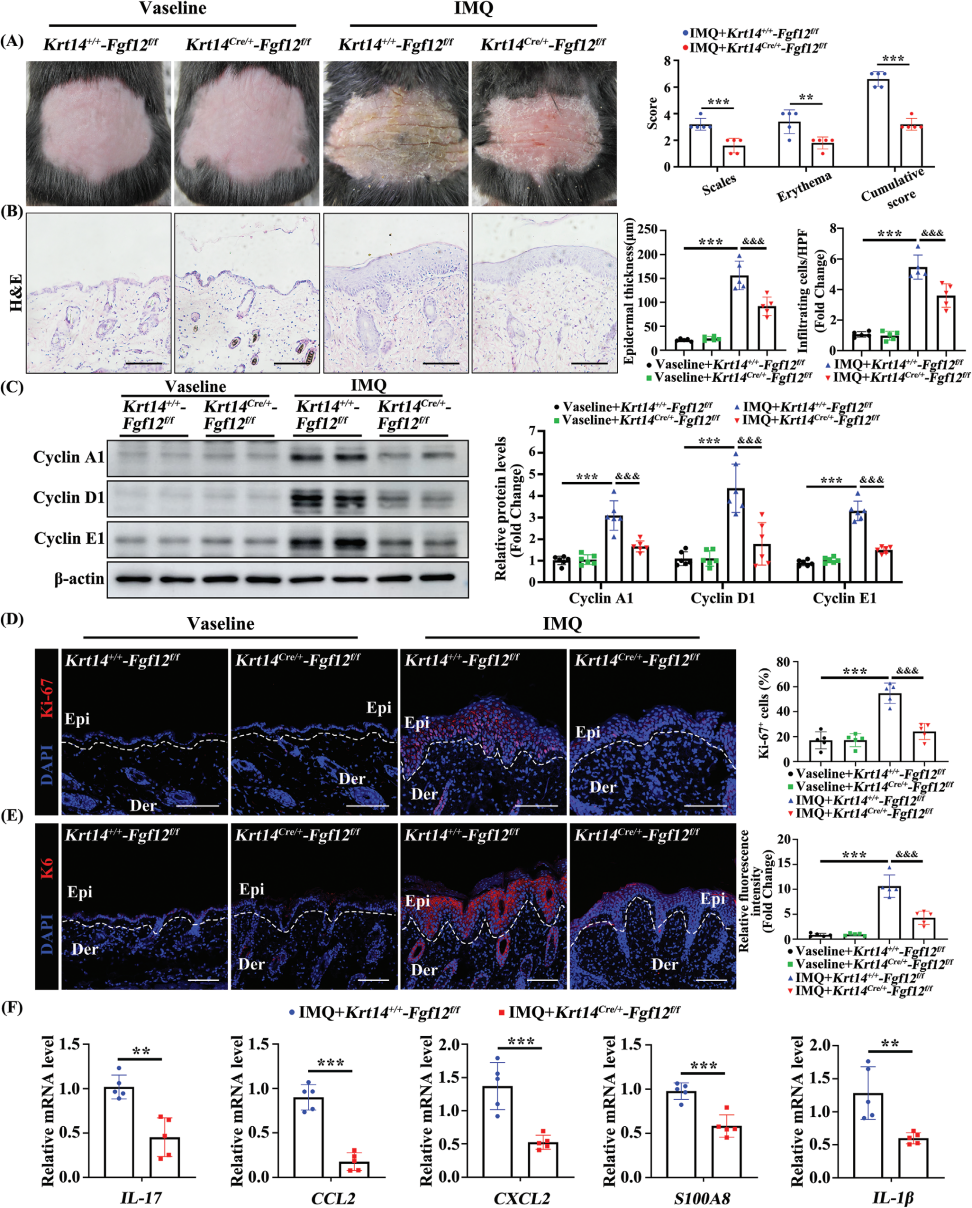
FGF12 ablation in keratinocytes ameliorates the psoriasiform phenotype. A) Representative images of the dorsal back from mice, and mice PASI scores were depicted (n = 5). B) Representative histological sections of the dorsal back from *Krt14^+/+^‐Fgf12^f/f^* and *Krt14^Cre/+^‐Fgf12^f/f^* mice treated by Vaseline or IMQ for 9 days stained with H&E, and quantification of the epidermal thickness and the infiltrating cells (n = 5). Scale bars = 100 µm. C) Immunoblotting analysis of Cyclin A1, Cyclin D1, and Cyclin E1 protein levels in *Krt14^+/+^‐Fgf12^f/f^* and *Krt14^Cre/+^‐Fgf12^f/f^* mice were treated with Vaseline or IMQ for 9 days. β‐Actin was used as a loading control (n = 6). D) Immunofluorescent and quantitative analysis of Ki‐67 positive cells in the skin from *Krt14^+/+^‐Fgf12^f/f^* and *Krt14^Cre/+^‐Fgf12^f/f^* mice were treated with Vaseline or IMQ for 9 days. Nuclei were stained with DAPI (blue) (n = 5). Scale bar = 100 µm. E) Immunofluorescent and quantitative analysis of K6 in the skin from *Krt14^+/+^‐Fgf12^f/f^* and *Krt14^Cre/+^‐Fgf12^f/f^* mice were treated with Vaseline or IMQ for 9 days. Nuclei were stained with DAPI (blue) (n = 5). Scale bar = 100 µm. F) qRT‐PCR analysis for *IL‐17*, *CCL2, CXCL2, S100A8* and *IL‐1β* mRNA levels in the skin from *Krt14^+/+^‐Fgf12^f/f^* and *Krt14^Cre/+^‐Fgf12^f/f^* mice were treated with IMQ for 9 days (n = 5). Error bars show the mean ± SEM. **p < 0.01; ***p < 0.001. *^&&&^ p* < 0.001. The *p* value was determined using two‐tailed unpaired Student's t test (A and F) or one‐way ANOVA (B‐E). All numbers (n) are biologically independent experiments.

To investigate whether FGF12 plays a positive or negative role in psoriasis, we crossed *Fgf12 floxed* mice (referred to as *Krt14^+/+^*‐*Fgf12^f/f^* mice hereafter) with *Krt14‐Cre* transgenic mice to selectively ablate of FGF12 in keratinocytes (referred to as *Krt14^Cre/+^*‐*Fgf12^f/f^* mice hereafter) and thereby determine the role of FGF12 in keratinocytes in psoriasis (Figure S2A,B, Supporting Information). Keratinocyte‐specific deletion of FGF12 was confirmed by immunoblotting (Figure S2C, Supporting Information). HE staining showed that specific knockout of FGF12 in keratinocytes did not significantly impact the thickness of the epidermal layer under normal conditions (Figure S2D, Supporting Information). This suggests that FGF12 does not play a crucial role in the regulation of the thickness of the epidermal layer in the absence of any external stimuli or pathological conditions. By contrast, the psoriasis area and severity index (PASI: evaluates erythema and scales) and epidermal thickness were higher on the backs of *Krt14^+/+^*‐*Fgf12^f/f^* mice than the *Krt14^Cre/+^*‐*Fgf12^f/f^* mice (**Figure** **2**A,B). Furthermore, there were more infiltrating cells on the backs of *Krt14^+/+^*‐*Fgf12^f/f^* mice than on the backs of *Krt14^Cre/+^‐Fgf12^f/f^* mice (Figure 2B). Next, we analyzed Cyclin A1, Cyclin D1, and Cyclin E1, which play a crucial role in the regulation of cell proliferation by functioning as important cell cycle regulators. These proteins were downregulated in *Krt14^Cre/+^‐Fgf12^f/f^* mice compared with *Krt14^+/+^‐Fgf12^f/f^* mice (Figure 2C). This finding suggests that the absence of FGF12 specifically in keratinocytes reduces expression of these proliferation‐related proteins, indicating that FGF12 promotes cell cycle progression in the epidermis. Consistently, the number of Ki‐67^+^ keratinocytes was markedly lower in IMQ‐treated *Krt14^Cre/+^‐Fgf12^f/f^* mice than *Krt14^+/+^‐Fgf12^f/f^* mice (Figure 2D).

In addition to its role in promoting proliferation, IMQ treatment induces accumulation of inflammatory cells in the skin and secretion of inflammatory factors. Therefore, we investigated the impact of FGF12 on IMQ‐induced skin inflammation by conducting immunofluorescence analysis to assess the expression levels of markers of skin inflammation and hyperproliferation, including K6 (Figure 2E). As expected, K6 expression was lower in IMQ‐treated *Krt14^Cre/+^‐Fgf12^f/f^* mice than *Krt14^+/+^‐Fgf12^f/f^* mice. To understand the influence of FGF12 on the inflammatory response associated with psoriasis development, we examined the gene expression levels of various inflammatory markers by qRT‐PCR. Expression of the T17 cell chemotactic factor *IL‐17*, neutrophil chemotactic factors (*CCL2* and *CXCL2*), the antimicrobial peptide *S100A8*, and *IL‐1β* in skin lesions was lower in *Krt14^Cre/+^‐Fgf12^f/f^* mice than in *Krt14^+/+^‐Fgf12^f/f^* mice (Figure 2F). These findings provide valuable insights into the potential role of FGF12 in the modulation of keratinocyte proliferation and inflammation in response to IMQ treatment and suggest that the absence of FGF12 specifically in keratinocytes alleviates skin inflammation and hyperproliferation in response to IMQ treatment.

### 2.3 FGF12 Is Required for Proliferation and Cell Cycle Transition of Keratinocytes

**Figure 3:**
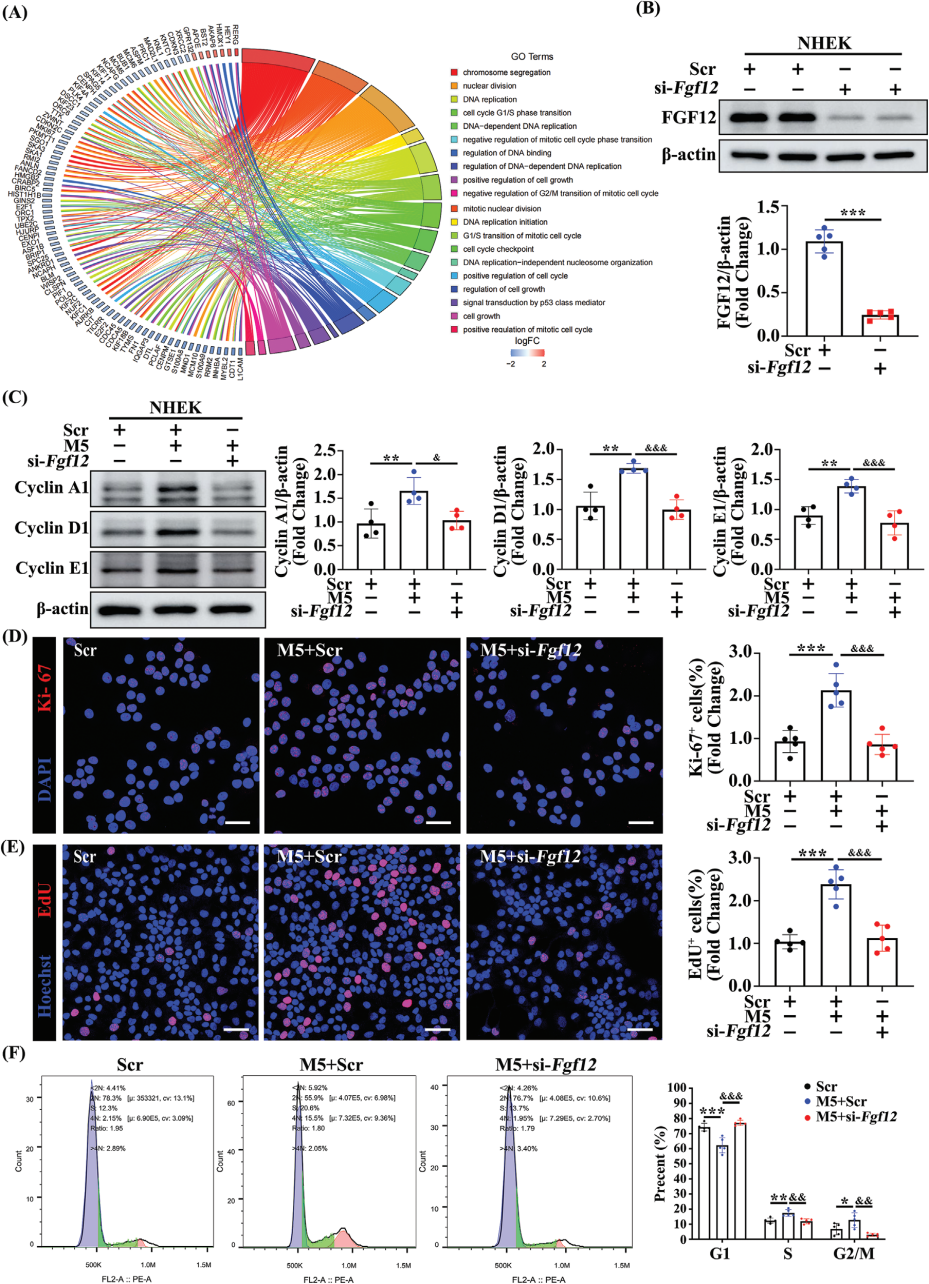
FGF12 is required for proliferation and cell cycle transition of keratinocytes. A) RNA‐Sequence analyzes the differentially regulated genes between si‐Scr group and si‐*Fgf12* group treated with M5 for 12 h. Chord diagram showing the top 20 enriched GO clusters. B) Immunoblotting and quantitative analysis of FGF12 protein level in NHEK cells that were transfected with si‐Scr or si‐*Fgf12*. β‐Actin was used as a loading control (n = 5). C) Immunoblotting and quantitative analysis of Cyclin A1, Cyclin D1, and Cyclin E1 protein levels in NHEK cells that were treated with si‐Scr or si‐*Fgf12* and stimulated with or without M5 for 12 h. β‐Actin was used as a loading control (n = 4). D) Immunofluorescent and quantitative analysis of Ki‐67^+^ in NHEK cells that were treated with si‐Scr or si‐*Fgf12* and stimulated with or without M5 for 12 h (n = 5). Scale bar = 50 µm. E) Immunofluorescent and quantitative analysis of EdU^+^ in HaCaT cells that were treated with si‐Scr or si‐*Fgf12* and stimulated with or without M5 for 12 h. Nuclei were stained with Hoechst (blue) (n = 5). Scale bar = 50 µm. F) Flow cytometric plots of cell‐cycle analysis performed with PI staining on HaCaT cells treated with si‐Scr or si‐*Fgf12* and stimulated with or without M5 for 12 h (left). Quantification the percentage of cells that fall into the sub G0/G1, S, or G2/M gates (right) (n = 5). Error bars show the mean ± SEM. *p < 0.05; **p < 0.01; ***p < 0.001. *^&^p* < 0.05; *^&&^p* < 0.01; *^&&&^ p* < 0.001. The *p* value was determined using two‐tailed unpaired Student's t test (B) or one‐way ANOVA (C‐F). All numbers (n) are biologically independent experiments.

The role of FGF12 in cell proliferation is well‐established,^[^ ^41^ ^]^ but its regulation and function in the context of psoriasis have not been thoroughly explored. Our findings demonstrated that FGF12 is present in psoriatic keratinocytes, indicating that it has a regulatory role in the function of these cells. Further investigations are needed to fully understand the specific functions of FGF12 in the skin and the underlying mechanisms, particularly in the context of psoriasis. To this end, we employed small interfering RNA (siRNA) to silence endogenous FGF12 expression in HaCaT cells (Figure S3A, Supporting Information) and then compared keratinocytes between the si‐*Fgf12*‐treated and control groups in the presence of M5 by performing RNA sequencing analysis. This allowed us to investigate the transcriptional changes and potential molecular pathways affected by silencing of FGF12 in keratinocytes, providing valuable insights into the functional role of FGF12 in these cells. Gene ontology enrichment analysis revealed that the silencing of FGF12 was associated with alterations of the cell cycle transition and proliferation of keratinocytes, which suggests that FGF12 plays an important role in the regulation of these processes in keratinocytes (**Figure** **3**A). To further investigate the effects of FGF12 depletion on cell cycle, we examined the protein expression levels of Cyclin A1, Cyclin D1, and Cyclin E1, which are markers of the G1/S cell cycle transition and proliferation. The expression levels of these proteins were significantly decreased in FGF12‐depleted keratinocytes, despite M5 treatment both in NHEK and HaCaT cells (Figure 3B,C; Figure S3B, Supporting Information). These results provide further evidence that FGF12 modulates proliferation and the G1/S cell cycle transition of keratinocytes. Furthermore, to assess the impact of FGF12 knockdown on cell proliferation, we performed Ki‐67 staining, which allows visualization of actively proliferating cells. The number of Ki‐67^+^ cells was significantly lower in the si‐*Fgf12*‐treated group than in the si‐Scr‐treated group upon M5 treatment (Figure 3D; Figure S3C, Supporting Information). Additionally, the number of cells positive for EdU, another marker of cell proliferation, was markedly lower in the si‐*Fgf12*‐treated group (Figure 3E). To gather further evidence supporting the role of FGF12 in promoting the G1/S cell cycle transition, we utilized flow cytometry to analyze the cell cycle distribution. The results showed that FGF12 deficiency resulted in cell cycle arrest in the G0/G1 phase (Figure 3F). Overall, these findings provide strong evidence that FGF12 depletion impairs proliferation and disrupts the G1/S cell cycle transition in keratinocytes.

Considering that increased proliferation is often associated with inhibition of apoptosis, we investigated whether FGF12 prevents apoptosis. As expected, flow cytometric analysis demonstrated that the percentage of Annexin V‐FITC^+^ apoptotic cells was significantly higher in the si‐*Fgf12*‐treated group than in the si‐Scr‐treated group (Figure S4A, Supporting Information). TUNEL staining provided further evidence that silencing of FGF12 increased apoptosis (Figure S4B, Supporting Information). These findings were confirmed by western blot analysis, which showed that expression of BCL2, an anti‐apoptotic marker, was downregulated and expression of cleaved caspase‐3 (C‐CAS‐3), a pro‐apoptotic marker, was upregulated in the si‐*Fgf12*‐treated group, particularly upon treatment with M5 (Figure S4C, Supporting Information). Moreover, we found that knockout of FGF12 specifically in mice keratinocytes induced apoptosis and inhibited aberrant proliferation of keratinocytes, which ultimately reduced the severity of psoriasis (Figure S4D, Supporting Information). Taken together, these results suggest that FGF12 promotes proliferation and cell cycle transition, and inhibits apoptosis of keratinocytes.

Consistently, in vitro experiments showed that overexpression of FGF12 was strongly associated with the upregulation of cell cycle‐related proteins involved in cell proliferation. Specifically, the expression levels of Cyclin A1, Cyclin D1, and Cyclin E1 were higher in HaCaT cells overexpressing FGF12 compared to those in Vector control HaCaT cells (Figure S5A, Supporting Information). Similarly, flow cytometric analysis revealed that overexpression of FGF12 accelerated the transition from G1/S phase to G2/M phase of the cell cycle and thereby stimulated cell proliferation (Figure S5B, Supporting Information). Immunofluorescence staining of EdU and Ki‐67 showed that the presence of FGF12 in conjunction with M5 treatment significantly enhanced the proliferation of keratinocytes (Figure S5C,D, Supporting Information). In conclusion, these findings collectively indicate that FGF12 plays a crucial role in the pathogenesis of psoriasis by influencing the cell cycle and facilitating proliferation of keratinocytes.

### 2.4 FGF12 Promotes Proliferation and Cell Cycle Transition of Keratinocytes through p53 Signaling

**Figure 4:**
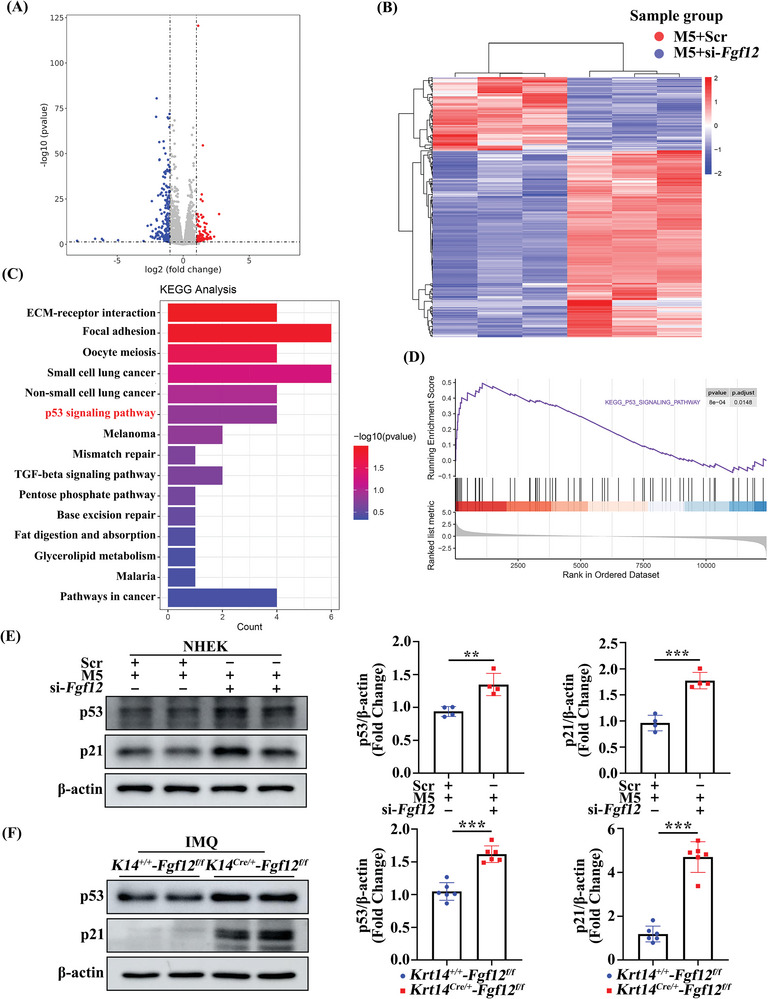
FGF12 promotes proliferation and cell cycle transition of keratinocytes through p53 signaling. A–C) KEGG analysis for the significantly upregulated signaling by the interference of si‐Scr or si‐*Fgf12* in HaCaT cells treated by M5 for 12 h. The Top 15 upregulated GO signal pathways were listed. D) GSEA showing the significant enrichment of p53 signaling in M5 treated HaCaT cells under FGF12 interference. E) Immunoblotting and quantitative analysis of p53 and p21 protein levels in NHEK cells that were treated with si‐Scr or si‐*Fgf12* and stimulated with M5 for 12 h. β‐Actin was used as a loading control (n = 4). F) Immunoblotting and quantitative analysis of p53 and p21 levels in *Krt14^+/+^‐Fgf12^f/f^* and *Krt14^Cre/+^‐Fgf12^f/f^* mice were treated with IMQ. β‐Actin was used as a loading control (n = 6). Error bars show the mean ± SEM. **p < 0.01; ***p < 0.001. The *p* value was determined using two‐tailed unpaired Student's t test (E and F). All numbers (n) are biologically independent experiments.

To investigate the molecular mechanisms by which FGF12 regulates keratinocyte proliferation, we conducted RNA sequencing analysis of M5‐treated keratinocytes (**Figure** **4**A,B). KEGG pathway enrichment analysis revealed that FGF12 silencing upregulated the p53 signaling pathway, suggesting that FGF12 increases keratinocyte proliferation by inhibiting this pathway (Figure 4C). Additionally, gene set enrichment analysis consistently demonstrated that the p53 signaling pathway was enriched in FGF12‐depleted keratinocytes (Figure 4D).

Western blot analysis revealed that the expression level of p53 was decreased in M5‐treated keratinocytes and the epidermis of IMQ‐treated psoriasis mice (Figure S6A,B, Supporting Information). Upon M5 treatment, FGF12 knockdown significantly induced expression of p53 and p21 (Figure 4E; Figure S6C, Supporting Information), while FGF12 overexpression had the opposite effect (Figure S6D, Supporting Information). These results indicate that FGF12 inhibits the p53 signaling pathway and thereby increases keratinocyte proliferation. Furthermore, qRT‐PCR demonstrated that FGF12 overexpression reduced the mRNA levels of *p53* and *p21* (Figure S6E, Supporting Information). FGF12 overexpression also suppressed the luciferase activity of p53 (Figure S6F, Supporting Information). Immunofluorescence analysis showed that FGF12 knockdown in keratinocytes upregulated the expression levels of p53 and p21 in the nucleus (Figure S6G,H, Supporting Information). Last, western blot analysis of the mouse model further confirmed that targeted knockout of FGF12 in keratinocytes activated the p53 signaling pathway (Figure 4F). In summary, these results indicate that FGF12 inhibits p53 activation to increase keratinocyte proliferation.

### 2.5 FGF12 Deficiency‐Driven Amelioration of Keratinocyte Proliferation Is p53 Dependent

**Figure 5:**
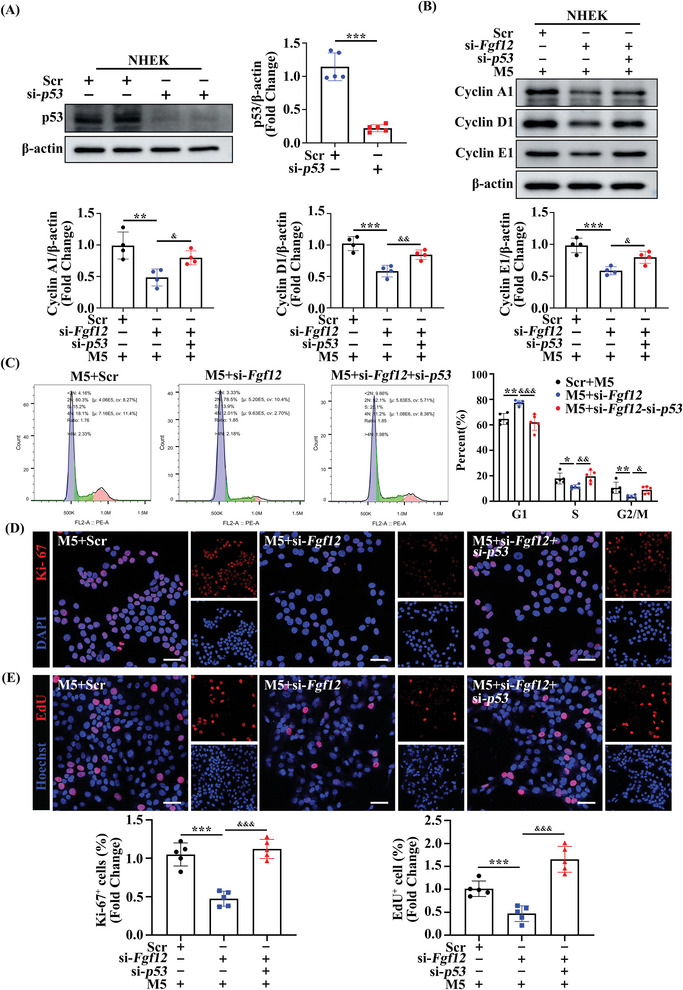
FGF12 deficiency‐driven amelioration of keratinocyte proliferation is p53 dependent. A) Immunoblotting and quantitative analysis of p53 protein level in NHEK cells that were transfected in si‐Scr or si‐*p53*. β‐Actin was used as a loading control (n = 5). B) Immunoblotting and quantitative analysis of Cyclin A1, Cyclin D1, and Cyclin E1 protein levels in NHEK cells that si‐*p53* or si‐Scr was transfected in FGF12‐interference cells treated with M5 for 12 h. β‐Actin was used as a loading control (n = 4). C) Flow cytometric plots of cell‐cycle analysis performed with PI staining on HaCaT cells that si‐*p53* or si‐Scr was transfected in FGF12‐interference cells treated with M5 for 12 h (left). Quantification the percentage of cells that fall into the sub G0/G1, S, or G2/M gates (right) (n = 5). D) Immunofluorescent and quantitative analysis (bottom) of Ki‐67^+^ in NHEK cells that si‐*p53* or si‐Scr was transfected in FGF12‐interference cells treated with M5 for 12 h. Nuclei were stained with DAPI (blue) (n = 5). Scale bar = 50 µm. E) Immunofluorescent and quantitative analysis (bottom) of EdU^+^ in HaCaT cells that si‐*p53* or si‐Scr was transfected in FGF12‐interference cells treated with M5 for 12 h. Nuclei were stained with Hoechst (blue) (n = 5). Scale bar = 50 µm. Error bars show the mean ± SEM. *p < 0.05; **p < 0.01; ***p < 0.001. *^&^ p* < 0.05; *^&&^p* < 0.01; *^&&&^p* < 0.001. The *p* value was determined using two‐tailed unpaired Student's t test (A) or one‐way ANOVA (B‐E). All numbers (n) are biologically independent experiments.

To investigate the role of p53 in the proliferative effect of FGF12 in psoriasis, we transfected p53‐targeting siRNA into FGF12‐deficient keratinocytes. Western blot assays were performed to confirm the successful knockdown of p53 expression (**Figure** **5**A, S7A, Supporting Information). The reduced expression of the cell cycle‐related proteins Cyclin A1, Cyclin D1, and Cyclin E1 upon FGF12 knockdown was reversed by p53 depletion (Figure 5B; Figure S7B, Supporting Information). We performed flow cytometry to validate the mitigatory effect of p53 knockdown on cell cycle blockade caused by FGF12 depletion. Blockade in the G0/G1 phase was relieved in keratinocytes lacking both p53 and FGF12 compared with those lacking only FGF12 (Figure 5C). Moreover, Ki‐67 and EdU staining revealed that p53 knockdown reversed the inhibitory effect of FGF12 knockdown on the proliferation of keratinocytes, indicating that p53 plays a crucial role in the regulation of cell proliferation by FGF12 (Figure 5D,E; Figure S7C, Supporting Information). In summary, these results confirm the conclusion that FGF12 increases keratinocyte proliferation in psoriasis primarily by inactivating the p53 signaling pathway.

### 2.6 FGF12 Represses Expression of p53 through MDM2

**Figure 6:**
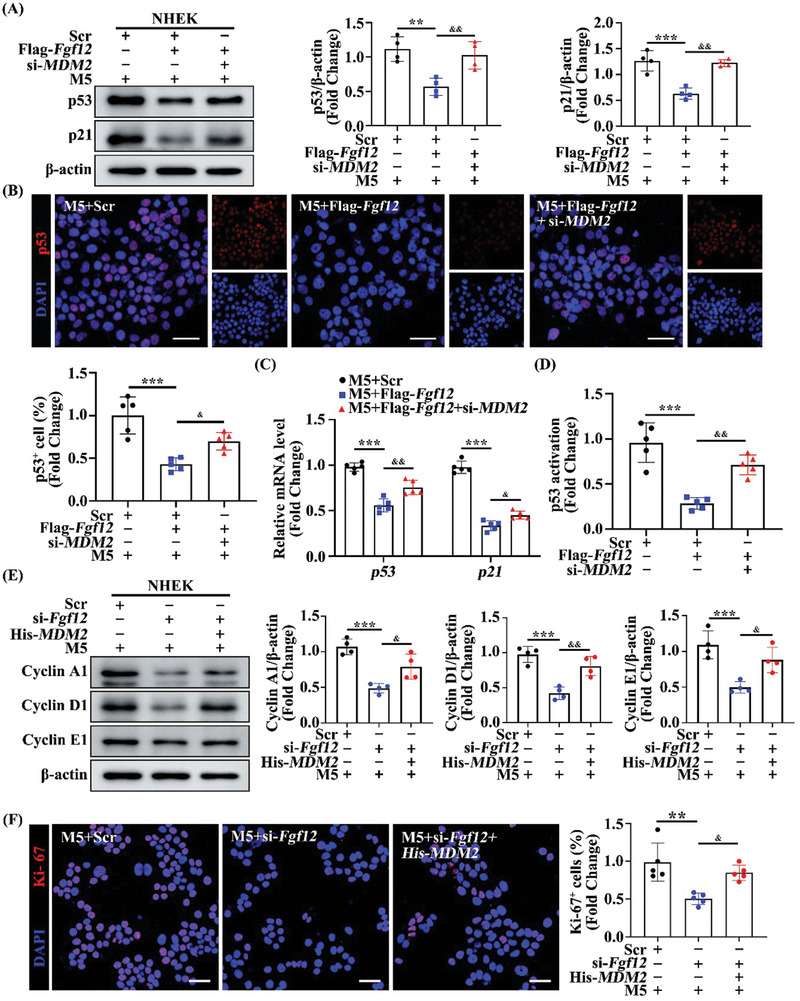
FGF12 represses expression of p53 through MDM2. A) Immunoblotting and quantitative analysis of p53 and p21 protein levels in NHEK cells that si‐*MDM2* or si‐Scr was transfected in Flag‐*Fgf12* cells treated with M5. β‐Actin was used as a loading control (n = 4). B) Immunofluorescent and quantitative analysis (down) of p53 in HaCaT cells that si‐*MDM2* or si‐Scr was transfected in Flag‐*Fgf12* cells treated with M5. Nuclei were stained with DAPI (blue) (n = 5). Scale bar = 50 µm. C) qRT‐PCR analysis for *p53* and *p21* mRNA levels in HaCaT cells that si‐*MDM2* or si‐Scr was transfected in Flag‐*Fgf12* cells treated with M5 for 12 h (n = 5). D) p53‐dependent transcriptional activity of p53 determined by performing dual‐luciferase assays with HEK293 cells overexpressing FGF12 in the presence of si‐Scr, or si‐*MDM2* (n = 5). E) Immunoblotting and quantitative analysis of Cyclin A1, Cyclin D1, and Cyclin E1 protein levels in NHEK cells that Vector or His‐*MDM2* was transfected in FGF12‐interference cells treated with M5 for 12 h. β‐Actin was used as a loading control (n = 4). F) Immunofluorescent and quantitative analysis of Ki‐67^+^ in NHEK cells that Vector or His‐*MDM2* was transfected in FGF12‐interference cells treated with M5 for 12 h. Nuclei were stained with DAPI (blue) (n = 5). Scale bar = 50 µm. Error bars show the mean ± SEM. **p < 0.01; ***p < 0.001. ^&^ *p* < 0.05; ^&&^ *p* < 0.01. The *p* value was determined using one‐way ANOVA (A‐F). All numbers (n) are biologically independent experiments.

MDM2, a classical regulatory factor of p53, functions as an E3 ubiquitin ligase that promotes ubiquitination and degradation of p53 and thereby inhibits p53 signaling. Interestingly, mutations of MDM2 enhance transcription of p53 target genes, suggesting that MDM2 also regulates p53 activity at the transcriptional level.^[^ ^31^ ^]^ To investigate the mechanism by which FGF12 regulates the p53 signaling pathway in psoriasis and keratinocytes, we assessed the protein levels of p53 and p21 in keratinocytes overexpressing FGF12 in the presence or absence of si‐*MDM2*. The protein levels of p53 and p21 were significantly decreased in keratinocytes overexpressing FGF12 but were substantially upregulated upon depletion of MDM2 (**Figure** **6**A; Figure S8A, Supporting Information). Immunofluorescence staining revealed that inhibition of the p53 signaling pathway induced by FGF12 was effectively blocked by depletion of MDM2 in HaCaT cells (Figure 6B). We assessed the mRNA levels of key components of the p53 signaling pathway. Expression of *p53* and *p21* was significantly increased upon MDM2 deletion despite overexpression of FGF12 (Figure 6C). Furthermore, we performed a luciferase reporter gene assay to measure the transcriptional activity of p53. As expected, FGF12 significantly inhibited p53 transcriptional activity; however, this effect was abolished by depletion of MDM2 (Figure 6D). Overall, these results suggest that MDM2 plays a crucial role in mediating the inhibitory effect of FGF12 on p53 signaling activity.

To further investigate the potential role of FGF12 and MDM2 in keratinocyte proliferation, we assessed the proteins levels of Cyclin A1, Cyclin D1, and Cyclin E1 in NHEK cells and observed that MDM2 overexpression partially counteracted the inhibitory effects of FGF12 deficiency on keratinocyte proliferation (Figure 6E; Figure S8B, Supporting Information). Similarly, we performed immunofluorescence staining of Ki‐67 and EdU, and the results showed that the levels of Ki‐67^+^ and EdU^+^ keratinocytes were markedly decreased upon FGF12 knockdown and these effects were largely reversed by overexpression of MDM2, indicating that MDM2 plays a positive role in the effects of FGF12 on proliferation of keratinocytes (Figure 6F; Figure S8C,D, Supporting Information). In summary, our in vitro experiments indicate that MDM2 is essential for the inhibitory effect of FGF12 on p53 activity during psoriasis, probably due to its suppressive effect on the transcription of p53.

### 2.7 FGF12 Stabilizes MDM2 by Hindering Its K48‐Linked Ubiquitination

**Figure 7:**
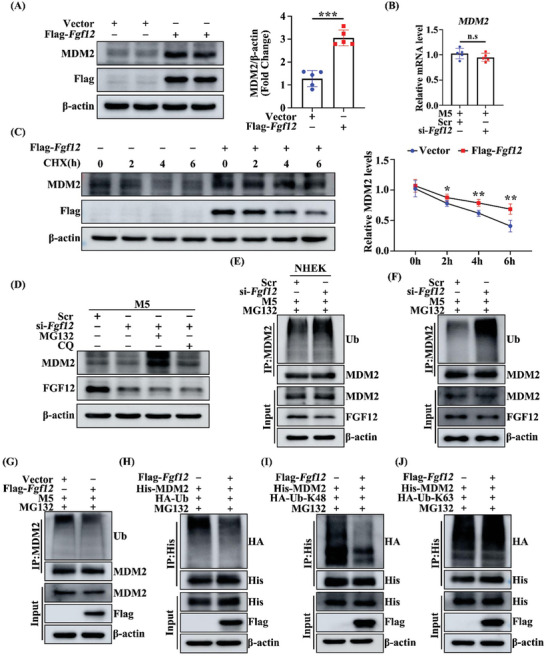
FGF12 stabilizes MDM2 by hindering its K48‐linked ubiquitination. A) Immunoblotting and quantitative analysis of MDM2 protein level that Flag‐*Fgf12* or Vector were transfected in HEK293 cells. β‐Actin was used as a loading control (n = 5). B) qRT‐PCR analysis for *MDM2* mRNA level that si‐Scr or si‐*Fgf12* were transfected in HEK293 cells treated with M5 for 12 h (n = 5). C) Immunoblotting and quantitative analysis of MDM2 protein levels that Flag‐*Fgf12* or Vector was transfected in HEK293 cells treated with cyclohexamide (CHX) for 0, 2, 4, and 6 h. β‐Actin was used as a loading control (n = 5). D) Immunoblotting of MDM2 protein level that si‐Scr or si‐*Fgf12* were transfected in HaCaT cells treated with MG132 or CQ for 6 h. β‐Actin was used as a loading control. E) The NHEK cells were transfected with si‐Scr and si‐*Fgf12* and then treated with M5 for 12 h. Cells were treated with MG132 (10 µM) for 6 h before lysation. The cell lysates were immunoprecipitated by anti‐MDM2 antibody, and then western blot assay with anti‐Ub, anti‐FGF12, and anti‐MDM2 antibody. F) The HaCaT cells were transfected with si‐Scr and si‐*Fgf12* and then treated with M5 for 12 h. Cells were treated with MG132 (10 µM) for 6 h before lysation. The cell lysates were immunoprecipitated by anti‐MDM2 antibody, and then western blot assay with anti‐Ub, anti‐FGF12, and anti‐MDM2 antibody. G) The HaCaT cells were transfected with Vector and Flag‐*Fgf12* and then treated with M5 for 12 h. Cells were treated with MG132 (10 µM) for 6 h before lysation. The cell lysates were immunoprecipitated by anti‐MDM2 antibody, and then western blot assay with anti‐Ub, anti‐Flag, and anti‐MDM2 antibody. H) The HEK293 cells were co‐transfected with Flag‐*Fgf12*, His‐*MDM2*, and HA‐*Ub* plasmids. Cells were treated with MG132 (10 µM) for 6 h before lysation. The cell lysates were immunoprecipitated by anti‐His antibody, and then western blot assay with anti‐HA, anti‐His, and anti‐Flag antibody. I) The HEK293 cells were co‐transfected with Flag‐*Fgf12*, His‐*MDM*2, and HA‐*Ub‐K48* plasmids. Cells were treated with MG132 (10 µM) for 6 h before lysation. The cell lysates were immunoprecipitated by anti‐His antibody, and then western blot assay with anti‐HA, anti‐His, and anti‐Flag antibody. J) The HEK293 cells were co‐transfected with Flag‐*Fgf12*, His‐*MDM2*, and HA‐*Ub‐K63* plasmids. Cells were treated with MG132 (10 µM) for 6 h before lysation. The cell lysates were immunoprecipitated by anti‐His antibody, and then western blot assay with anti‐HA, anti‐His, and anti‐Flag antibody. Error bars show the mean ± SEM. n.s., not significant; *p < 0.05; **p < 0.01; ***p < 0.001. The *p* value was determined using two‐tailed unpaired Student's t test (A and B) or one‐way ANOVA (C). All numbers (n) are biologically independent experiments.

To investigate the impact of FGF12 on MDM2 expression, we conducted experiments in keratinocytes and HEK293 cells. Knockdown of FGF12 significantly decreased MDM2 protein expression, particularly upon treatment with M5 in HaCaT cells (Figure S9A, Supporting Information). Similarly, overexpression of FGF12 in HEK293 cells upregulates the expression of MDM2 (**Figure** **7**A).

Silencing of FGF12 did not change the mRNA level of MDM2, suggesting that FGF12 regulates MDM2 protein expression at the post‐translational level (Figure 7B). To confirm this, we examined the effect of FGF12 on the stability of MDM2. A cycloheximide chase assay indicated that overexpression of FGF12 delayed the degradation of MDM2 protein (Figure 7C). The proteasome inhibitor MG132 attenuated the effect of FGF12 knockdown on the MDM2 protein level, but the autophagic lysosome inhibitor chloroquine did not, indicating that FGF12 sustains MDM2 stability through the ubiquitination‐proteasome pathway (Figure 7D). Knockdown of FGF12 increased polyubiquitination of MDM2 in M5‐treated keratinocytes, whereas overexpression of FGF12 had the opposite effect (Figure 7E–G). Additionally, overexpression of FGF12 downregulated ubiquitin chains attached to MDM2 (Figure 7H). These results suggest that FGF12 decreases ubiquitination and subsequent degradation of MDM2.

To explore which type of MDM2 ubiquitination is regulated by FGF12, we co‐transfected HEK293 cells with Flag‐*Fgf12* and plasmids containing K48 or K63 ubiquitin. Immunoprecipitation (IP) showed that FGF12 significantly restrained K48‐linked, but not K63‐linked, ubiquitination of MDM2 (Figure 7I,J). This indicates that FGF12 reduces K48‐linked ubiquitination of MDM2 and thereby stabilizes this protein.

### 2.8 FGF12 Interacts with MDM2 to Block Binding of β‐Trcp to MDM2

**Figure 8:**
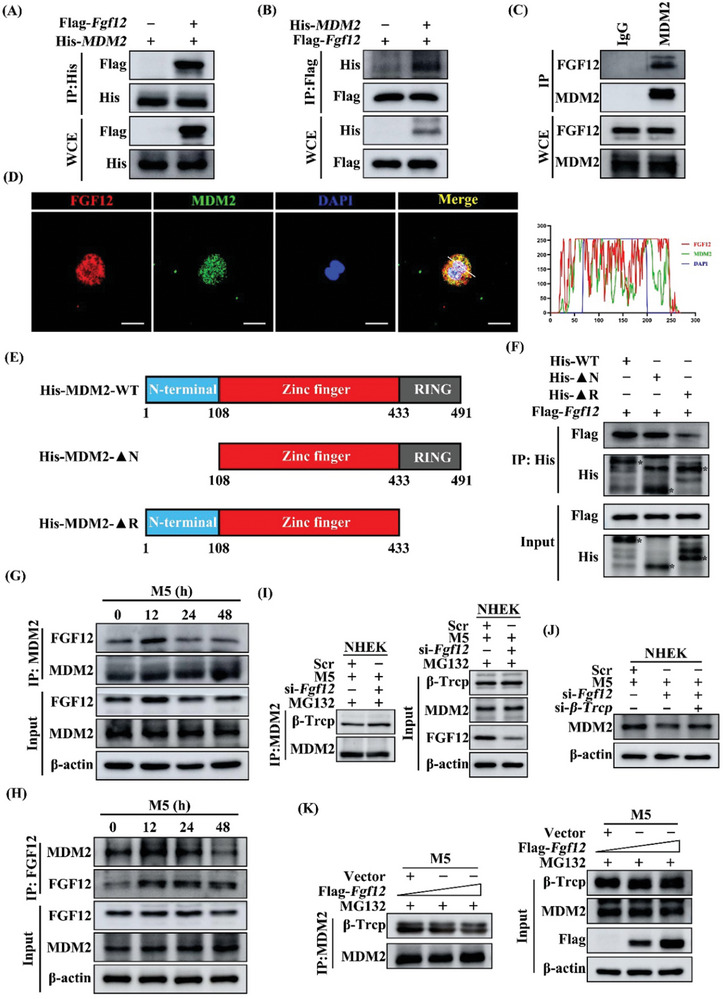
FGF12 interacts with MDM2 to block binding of β‐Trcp. A,B) The HEK293 cells were co‐transfected with Flag‐*Fgf1*2 and His‐*MDM*2 plasmids for 48 h. The lysates of cells were immunoprecipitated with His‐tag antibody and then western blot assay with Flag‐tag antibody (A); immunoprecipitated with Flag‐tag antibody and immunoblotted with His‐tag antibody (B). C) The HaCaT cell lysates were immunoprecipitated with IgG and anti‐MDM2 antibody and the expression of FGF12 and MDM2 were detected by western blot. D) Immunofluorescent staining (left) of FGF12 and MDM2 in HaCaT cells. Nuclei were stained with DAPI (blue). Scale bar = 20 µm. White lines in merged images (right) indicate the area where the distances between FGF12, MDM2, and DAPI were analyzed using ImageJ. E) The schematic diagram showed structural domains of MDM2 protein. F) HEK293 cells were transfected with Flag‐*Fgf12* and several MDM2 deletion mutants. The whole‐cell lysates were immunoprecipitated with anti‐His beads and immunoblotted with anti‐Flag and anti‐His antibodies. G) HaCaT cells and stimulated with M5 for 0, 12, 24, 48 h. Whole‐cell lysates were IP with anti‐MDM2 then subjected to immunoblot analysis with the anti‐FGF12, anti‐MDM2 antibodies. H) HaCaT cells and stimulated with M5 for 0, 12, 24, 48 h. Whole‐cell lysates were immunoprecipitated with anti‐FGF12 then subjected to immunoblot analysis with the anti‐FGF12, anti‐MDM2 antibodies. I) NHEK cells were transfected with si‐Scr and si‐*Fgf12* and then stimulated with M5 for 12 h. Cells were treated with MG132 (10 µM) for 6 h before lysation. Whole‐cell lysates were immunoprecipitated with anti‐MDM2 then subjected to immunoblot analysis with the anti‐β‐Trcp, anti‐MDM2 and anti‐FGF12 antibodies. J) Immunoblotting analysis of MDM2 protein level in NHEK cells that were transfected with si‐Scr, si‐*β‐Trcp* and si‐*Fgf12* treatment by M5 for 12 h. β‐Actin was used as a loading control. K) HaCaT cells were co‐transfected with Flag‐*Fgf12* plasmid (0, 2, 4 µg) and then stimulated with M5 for 12 h. Cells were treated with MG132 (10 µM) for 6 h before lysation. Whole‐cell lysates were immunoprecipitated with anti‐MDM2 then subjected to immunoblot analysis with the anti‐β‐Trcp, anti‐MDM2 and anti‐Flag antibodies. Data are representative of three independent experiments.

To determine the potential association between FGF12 and MDM2, we utilized the HDOCK server to perform protein docking analysis and found that FGF12 can interact with MDM2 (Figure S10A, Supporting Information). This interaction provides further insight into the molecular mechanism by which FGF12 regulates MDM2 stability. To validate this interaction, HEK293 cells were co‐transfected with Flag‐*Fgf12* and His‐*MDM2* and then subjected to a reciprocal co‐IP assay. This indicated that FGF12 interacted with MDM2 in HEK293 cells (**Figure** **8**A,B). A similar result was observed in HaCaT cells under physiological conditions (Figure 8C). This was confirmed by immunofluorescence staining (Figure 8D). MDM2 consists of three domains: an N‐terminal domain (amino acids 1–108), a zinc finger domain (amino acids 109–432), and a ring finger domain (amino acids 433–491). To identify which domain of MDM2 is essential for its binding with FGF12, we generated several truncation mutants of MDM2 (Figure 8E). Co‐IP showed that His‐MDM2‐WT and His‐MDM2‐ΔN bound to Flag‐FGF12, while binding of His‐MDM2‐ΔR to Flag‐FGF12 was reduced (Figure 8F). Based on these findings, we conclude that the RING domain of MDM2 is essential for its association with FGF12.

Furthermore, IP revealed that the binding of FGF12 to MDM2 was increased upon M5 stimulation for 12 h and exhibited time dependency (Figure 8G,H). The abundance of MDM2 can be regulated by its auto‐ubiquitination pathway as well as the β‐Trcp‐dependent ubiquitination pathway. We conducted IP experiments to investigate whether the degradation of MDM2 is mediated by β‐Trcp or its endogenous ubiquitin ligase activity. Depletion of FGF12 reduced the interaction between MDM2 and 14‐3‐3σ, which is key for auto‐degradation of MDM2 (Figure S10B, Supporting Information), indicating that FGF12 increases the stability of MDM2 independent of its auto‐ubiquitination degradation pathway. By contrast, the depletion of FGF12 significantly increased the interaction between β‐Trcp and MDM2 in NHEK and HaCaT cells (Figure 8I; Figure S10C, Supporting Information). Moreover, β‐Trcp knockdown restored the protein content of MDM2, even in the presence of si‐*Fgf12* in **keratinocyte**s (Figure 8J; Figure S10D–F, Supporting Information). These findings indicate that FGF12 inhibits degradation of MDM2 by blocking its interaction with β‐Trcp.

To confirm our conclusion, we performed IP with an anti‐MDM2 antibody in FGF12‐overexpressing HaCaT cells. As expected, the binding of β‐Trcp to MDM2 was decreased upon overexpression of FGF12 (Figure 8K). A similar result was observed in HEK293 cells (Figure S10G, Supporting Information). Taken together, our results indicate that FGF12 disrupts the interaction between MDM2 and β‐Trcp, which increases the stability of MDM2, and thereby inhibits the activity of p53 signaling pathway.

### 2.9 Loss of p53 Abolishes the Mitigatory Effects of FGF12 Knockdown on Psoriasis in Mice

**Figure 9:**
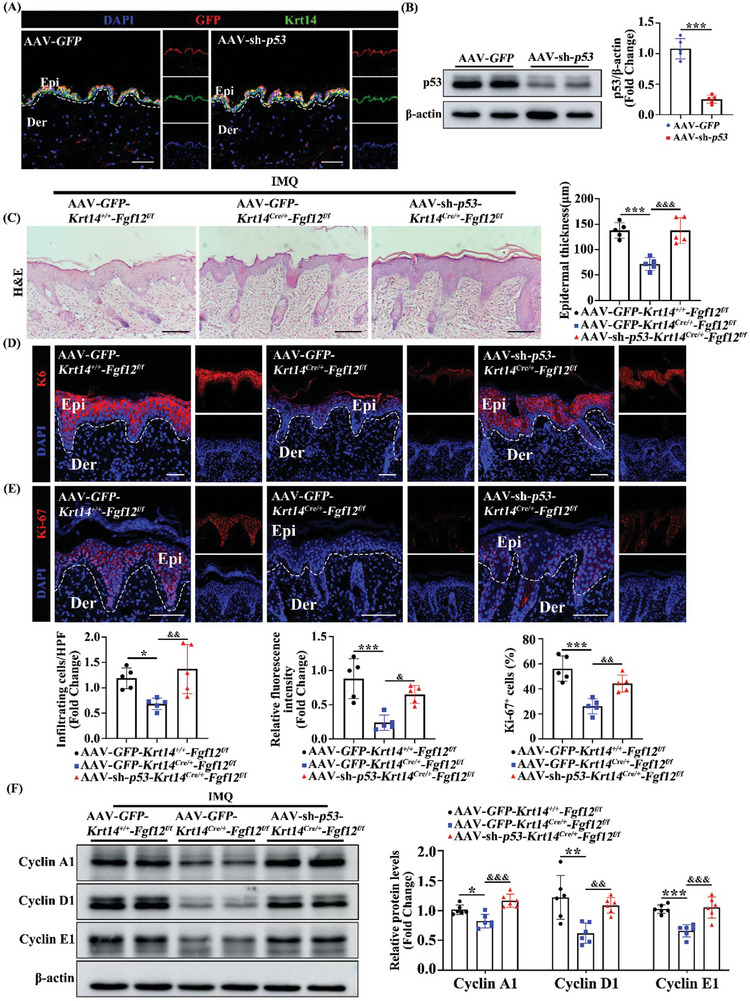
Loss of p53 abolishes the mitigatory effects of FGF12 knockdown on psoriasis in mice. A) Immunofluorescence images for the skin of mice with knock‐down respective genes were labeled with the indicated antibodies. Nuclei were stained with DAPI (blue). Scar bar = 50 µm. B) Immunoblotting and quantitative analysis of p53 protein level in AAV‐*GFP* and AAV‐sh‐*p53* mice. β‐Actin was used as a loading control (n = 5). C) Representative histological sections of the dorsal back from *Krt14^+/+^‐Fgf12^f/f^*; AAV‐*GFP*, *Krt14^Cre/+^‐Fgf12^f/f^*; AAV‐*GFP* and *Krt14^Cre/+^‐Fgf12^f/f^*; AAV‐sh‐*p53* mice treated by IMQ stained with H&E, and quantification of the epidermal thickness and the infiltrating cells (n = 5). Scale bars = 100 µm. D) Immunofluorescent and quantitative analysis of K6 in the skin from *Krt14^+/+^‐Fgf12^f/f^*; AAV‐*GFP*, *Krt14^Cre/+^‐Fgf12^f/f^*; AAV‐*GFP* and *Krt14^Cre/+^‐Fgf12^f/f^*; AAV‐sh‐*p53* mice induced by IMQ. Nuclei were stained with DAPI (blue) (n = 5). Scale bars = 50 µm. E) Immunofluorescent and quantitative analysis of Ki‐67 positive cells in the skin from *Krt14^+/+^‐Fgf12^f/f^*; AAV‐*GFP*, *Krt14^Cre/+^‐Fgf12^f/f^*; AAV‐*GFP* and *Krt14^Cre/+^‐Fgf12^f/f^*; AAV‐sh‐*p53* mice stimulated by IMQ. Nuclei were stained with DAPI (blue) (n = 5). Scale bars = 100 µm. F) Immunoblotting of Cyclin A1, Cyclin D1, and Cyclin E1 protein levels in the skin from *Krt14^+/+^‐Fgf12^f/f^*; AAV‐*GFP*, *Krt14^Cre/+^‐Fgf12^f/f^*; AAV‐*GFP* and *Krt14^Cre/+^‐Fgf12^f/f^*; AAV‐sh‐*p53* mice treated by IMQ. β‐Actin was used as a loading control (n = 6). Error bars show the mean ± SEM. *p < 0.05; **p < 0.01; ***p < 0.001. *^&^p* < 0.05; *^&&^p* < 0.01; *^&&&^p* < 0.001. The *p* value was determined using two‐tailed unpaired Student's t test (B) or one‐way ANOVA (C‐F). All numbers (n) are biologically independent experiments.

To investigate the impact of p53 on the positive effects of FGF12 in psoriasis, we used AAV‐mediated RNA interference to specifically knockdown p53 in keratinocytes (AAV9‐*Krt14‐sh‐p5*3) (**Figure** **9**A). The efficiency of p53 knockdown was confirmed by analyzing the level of p53 protein in the epidermis by western blotting (Figure 9B). HE staining revealed that specific knockout of FGF12 in keratinocytes decreased epidermal thickness and inflammatory cell infiltration. However, these effects were reversed when p53 was also knocked down (Figure 9C). Considering the crucial role of inflammation and proliferation in the development of psoriasis, we investigated if p53 plays a role in the regulation of epidermal keratinocytes by FGF12 in psoriasis. Immunofluorescence staining of K6 revealed that mice lacking FGF12 exhibited reduced inflammation and hyperproliferation of keratinocytes, and these effects were largely reversed by knockdown of p53 (Figure 9D). Immunofluorescence staining of Ki‐67 yielded consistent results, demonstrating that the reduction in keratinocyte proliferation observed in FGF12‐deficient mice was counteracted by p53 knockdown (Figure 9E). Moreover, FGF12 is closely associated with regulation of the cell cycle; therefore, the potential involvement of p53 in regulation of cell cycle‐related protein expression by FGF12 was explored in **mice**. Western blot analysis showed that decreased expression of Cyclin A1, Cyclin D1, and Cyclin E1 upon loss of FGF12 was partially restored by knockdown of p53 (Figure 9F). Based on these data, we believe that the p53 signaling pathway is essential for alleviation of the psoriasis phenotype upon loss of FGF12.

## 3 Discussion

Early studies of FGF12 predominantly investigated its functions in the nervous system, cardiovascular system, and liver. The role of FGF12 in the pathogenesis of psoriasis remains poorly understood. In this study, we aimed to investigate the abnormal expression of FGF12 in psoriatic keratinocytes and its potential role in the treatment of psoriasis. The specific knockout of FGF12 in keratinocytes reduced psoriatic symptoms in **mice**. Mechanistically, silencing of FGF12 expression in keratinocytes decreased cell proliferation, activated the p53 signaling pathway, and induced apoptosis. Our findings are significant because they demonstrate that FGF12 downregulates the activity of p53 signaling. We discovered that FGF12 competes with β‐Trcp for binding to MDM2, resulting in inhibition of K48‐linked ubiquitination of MDM2 and its subsequent stabilization. The interaction between FGF12 and MDM2 effectively hampers p53 signaling activity. These findings suggest that FGF12 plays a key role in abnormal proliferation of keratinocytes in psoriasis and could be a promising target for treatment of this disease.

Psoriasis is a chronic skin disease characterized by dysregulated innate and adaptive immune responses involving various cell types such as keratinocytes, dendritic cells, and T cells.^[^ ^42^ ^]^ Among these, keratinocytes are crucial because they contribute to both the initiation and perpetuation of abnormal proliferation and inflammation in psoriasis. Inhibition of keratinocyte proliferation alleviates psoriasis symptoms. For instance, topical application of a serine hydroxymethyltransferase inhibitor that suppresses keratinocyte proliferation in IMQ‐induced psoriasis models reduces expression of genes associated with psoriasis.^[^ ^43^ ^]^ In addition, berberine, which inhibits cell division cycle 6 expression and proliferation of human keratinocytes by interfering with the JAK‐STAT3 signaling pathway, is a potential treatment option for psoriasis patients.^[^ ^44^ ^]^ We observed significantly increased FGF12 expression in psoriatic lesions, particularly in the epidermis. The specific knockout of FGF12 in keratinocytes effectively reduced IMQ‐induced psoriatic‐like skin hyperplasia in mice. Furthermore, RNA sequencing analysis revealed that knockdown of FGF12 severely hindered the transition from G0/G1 phase of the cell cycle and cell proliferation. In addition, deletion of FGF12 in keratinocytes inhibited cell proliferation, decreased the proportions of Ki‐67^+^ and EdU^+^ cells, and reduced expression of Cyclin A1, Cyclin D1, and Cyclin E1. These findings provide further evidence that FGF12 is involved in the regulation of cell proliferation.

Although the role of the tumor suppressor protein p53 in skin cancers, such as melanoma and non‐melanoma, has been extensively studied, its role in psoriasis remains unclear. Known as the “guardian of the genome”, p53 acts as a transcription factor that regulates expression of various target genes involved in crucial cellular processes, including DNA repair, apoptosis, cell cycle, and differentiation, through its DNA‐binding domain.^[^ ^45^ ^]^ p53 is regulated by multiple external factors, with MDM2 being a key regulator. MDM2 not only mediates ubiquitination‐dependent degradation of p53, but also directly inhibits its transcriptional activity. We demonstrated that FGF12 inhibits the transcriptional activity of p53 and downregulates expression of its downstream target genes via MDM2, which induces cell cycle progression and cell proliferation.

Abnormal expression of MDM2 has been observed in various human cancers, potentially resulting from gene amplification, transcriptional regulation, and post‐translational modifications.^[^ ^46^ ^]^ Small molecule inhibitors of MDM2 have been investigated as potential therapies for cancer.^[^ ^47^ ^]^ At the protein level, the stability of MDM2 is regulated by other ubiquitin ligases, such as β‐Trcp, which directly binds to MDM2, leading to its degradation. Additionally, MDM2 possesses self‐ubiquitination capabilities, allowing self regulation, and binding of 14‐3‐3‐σ to MDM2 mediates its degradation. We revealed that FGF12 enhances the protein stability of MDM2. We found that FGF12 competes with β‐Trcp for binding to MDM2, thereby preventing β‐Trcp‐induced K48‐linked ubiquitination and subsequent degradation of MDM2. These findings shed light on a novel regulatory mechanism involving FGF12 and β‐Trcp that modulates the MDM2 protein level, and highlights the potential therapeutic relevance of targeting this interaction for diseases associated with MDM2 dysregulation, such as psoriasis.

The study also has some potential limitations. First, although we have performed some key experiments with primary keratinocytes, most of our cell‐level data were experimentally verified using HaCaT cells, which are immortalized epithelial cells with site mutations and greater proliferative capacity than primary cells. Second, we used IMQ‐induced psoriasis mouse model in this study, which is the most prevalent psoriatic mouse model due to its ease of use, convenience, and low cost. Nevertheless, the limitation of this model is that it can only be utilized as an acute model, which does not fully reproduce the chronic inflammatory state of psoriasis.^[^ ^48^, ^49^ ^]^ Consequently, further investigations are needed by using other psoriatic mouse models in the future.

In summary, the absence of FGF12 decreases the stability of MDM2 protein, triggering an increase in p53 transcriptional activity. This promotes cell cycle arrest and apoptosis of keratinocytes, and hinders progression of psoriasis. FGF12 is abundantly expressed in psoriasis and is found in close proximity to epidermal keratinocytes. Its loss leads to cell cycle arrest and reduced cell proliferation. Further investigations revealed that FGF12 bound to RING domain of MDM2 and prevented its β‐Trcp‐induced K48‐linked ubiquitination. This interaction stabilizes MDM2, resulting in inhibition of p53 transcriptional activity. The alleviatory effect of loss of FGF12 on psoriasis progression was reversed by p53 knockdown both in **mice** and in vitro. In conclusion, our study reveals that FGF12 positively regulates MDM2 expression in psoriatic keratinocytes and promotes progression of psoriasis by inhibiting p53 activity. These findings indicate that reducing FGF12 expression in keratinocytes could be a potential therapeutic approach for psoriasis.

## 4 Experimental Section

### Human Subjects

Psoriatic skin samples were obtained from the Department of Dermatology and Venereology, the First Affiliated Hospital of Wenzhou Medical University. All the patients were assessed according to the PASI. Normal adult human skin specimens were acquired from healthy donors who were undergoing plastic surgery. The study was performed in accordance with the principles of the Declaration of Helsinki and approved by the First Affiliated Hospital of Wenzhou Medical University (Institutional Review Board approval number KY2021‐106).

### Animals

The C57BL/6 mice, *Fgf12 flox/flox* mice (*Fgf12^fl/fl^*; C57BL/6 background), and *Krt14cre* mice (C57BL/6 background) used in this study were kept in a controlled environment that was free from pathogens. Male C57BL/6 mice were purchased from Shanghai Slac Laboratory Animal Co. Ltd. The facility maintained a temperature‐controlled setting with 12‐h cycles of light and darkness. The animals were provided with unlimited access to food and water. All experimental procedures and methods were approved by the Institutional Animal Care and Use Committee of Wenzhou Medical University.

### Adeno‐Associated Virus Production

AAV expressing GFP under the control of the skin epidermal keratinocyte‐specific *Krt14* promoter (Genechem, GOSV0207037_1): AAV9‐*Krt14 GFP* (AAV‐*GFP*) or AAV9‐*Krt14* sh‐*p53* (AAV‐sh‐*p53*) was injected subcutaneously using 35‐gauge needle around the specific region of dorsal skin. Five injections of ≈10 µL at a dose of 1.8 × 10^13^ viral genomes were administered. Three weeks after injection, AAV mediated gene knockdown was confirmed in the skin by immunofluorescence and western blot.

### Animal Model of Psoriasis and Treatment

The psoriasis animal model used in our research study was an Imiquimod (IMQ) (or Vaseline for control)‐induced psoriasis mouse model. Briefly, the day before induction, the backs of the mice were shaved and then treated with Aldara cream (Sichuan MingXin Pharmaceutical Co., LTD., H20030129) containing 5% IMQ (55 mg) once daily for 9 days.^[^ ^50^ ^]^ For the AAV‐sh‐*p53* experiments, following the three weeks injection, mice back skin daily prior to the IMQ treatment for 9 consecutive days. All procedures were approved and supervised by the Institutional Animal Care and Use Committee of Wenzhou Medical University.

### Cell Culture

The keratinocytes cell line HaCaT was obtained from the Wuhan (Procell, RRID: CVCL_0038), cultured in MEM medium (Procell, PM150478). Primary Normal Human Epidermal Keratinocytes (NHEK) were purchased from American Type Culture Collection (ATCC) (NHEK, PS‐200‐010, ATCC, Manassas, VA) and grown in dermal cell basal medium (PCS‐200‐030, ATCC). Human embryonic kidney cells (HEK293) were purchased from the ATCC (CRL‐1573) and cultured in Dulbecco's Modified Eagle's Medium (DMEM, Gibco). All cell media supplemented with 10% (vol/vol) fetal bovine serum (FBS; Thermo Fisher Scientific, 15140122). The cell culture conditions were 37°C and 5% CO_2_. All cells were found to be free from mycoplasma contamination.

### Induction of the In Vitro Psoriatic Model

Cells were stimulated with 10 ng mL^⁻1^ recombinant IL‐17A (Prospec Protein Specialists, CYT‐250), OSM (Prospec Protein Specialists, CYT‐231), TNF‐α (Prospec Protein Specialists, CYT‐223), IL‐22 (Prospec Protein Specialists, CYT‐328), and IL‐1α (Prospec Protein Specialists, CYT‐253) in combination (named M5) in medium supplemented with 2% (vol/vol) FBS to recapitulate numerous features of psoriasis.^[^ ^40^ ^]^

### Western Blot

RIPA lysis buffer (Thermo Fisher Scientific) containing Protease and Phosphatase Inhibitor Cocktail (Abcam, ab65621) was utilized to extract total proteins from fresh skin tissues and cell samples. The protein concentrations were determined using the Pierce BCA Protein Assay Kit (Thermo Fisher Scientific, 23228). The proteins were then separated by SDS‐PAGE and transferred onto a PVDF membrane (Millipore, IPVH00010). To prevent non‐specific binding, the membrane was blocked with 5% non‐fat milk (BD Biosciences, 232100). Subsequently, the membrane was incubated with primary antibodies followed by secondary antibodies. Finally, the blots were developed using the ECL reagent (Millipore, WBLUF0500) and captured using the Amersham Image 680 system (GE Healthcare Life Sciences). The antibodies used in the western blot analysis are listed in Table S1 (Supporting Information).

### RNA Isolation and Quantitative RT‐PCR

The total RNA from skin tissue or cells was extracted using TRIzol reagent (Thermo Fisher Scientific, 15596018) following the guidelines provided by the manufacturer. Reverse transcription was carried out using ReverTra Ace® qPCR RT Master Mix (Vazyme, Nanjing, China) as per the manufacturer's instructions. Real‐time PCR was performed using SYBR Green Master Mix (Vazyme, Nanjing, China). The mRNA expression levels of the analyzed genes were normalized and quantified using the 2^−ΔΔCt^ method. The primer sequences used for PCR amplification are provided in Table S2 (Supporting Information).

### RNA Interference

The experimental analysis was carried out after the siRNA transfection. For the negative control, siRNA obtained from Santa Cruz Biotechnology was used. Additionally, *Fgf12* siRNA (Santa Cruz Biotechnology, sc‐39466), *p53* siRNA (Santa Cruz Biotechnology, sc‐29435), *MDM*2 siRNA (Santa Cruz Biotechnology, sc‐29394), and *β‐Trcp* siRNA (Santa Cruz Biotechnology, sc‐37178) were utilized. The cells were seeded 12 h prior to transfection and allowed to reach a confluence of 30–50% by day 0. Then, 30 nM of the siRNA was transfected into the cells using Lipofectamine 3000 (Invitrogen, L3000‐001) and Opti‐MEMI Reduced Serum Medium (Gibco, 31 985 088), following the instructions provided by the manufacturers. On day 1, when the cells reached confluence (24 h after transfection), the siRNA solution was replaced with full growth medium. Subsequently, the experimental analysis was conducted.

### Histological Analysis

Paraffin‐embedded sections were stained using the Hematoxylin and Eosin kit (Solarbio, China) to assess the skin epidermal thickness. The sections were examined using a Nikon camera (Nikon, Melville, NY, USA).

### Immunofluorescence Staining Assay

For skin sections that were 5 µm thick, the process of deparaffinization and rehydration was conducted. Subsequently, antigen retrieval was performed by heating the slides in 10 mM Citrate buffer (pH 6.0) at 95 °C for 10 min. As for HaCaT and NHEK samples, the cells were fixed in 4% paraformaldehyde for 15 min and permeabilized with 0.5% Triton X‐100 for 15 min at room temperature. Following fixation and permeabilization, the samples were blocked with 5% (vol/vol) bovine serum albumin (BSA, Sigma‐Aldrich, B2064) for 1 h at room temperature. Next, the samples were incubated overnight at 4 °C with primary antibodies against FGF12 (SantaCruz Biotechnology, sc‐81947; 1:100 dilution), Ki‐67 (Cell Signaling Technology, 12 075; 1:50 dilution), Krt14 (Abcam, ab181595; 1:200 dilution), Cytokeratin 6A (K6) (Proteintech, 10590‐1‐AP; 1:400 dilution), p53 (Cell Signaling Technology, 2524; 1:2000 dilution), p21 (Cell Signaling Technology, 2947; 1:400 dilution), MDM2 (Abcam, ab259265; 1:100 dilution), and GFP (Abcam, ab183734; 1:500 dilution) followed by washing and incubation with secondary antibodies. Finally, DAPI was used to label the cell nuclei. Images were acquired using a Leica SP8 confocal microscope (Leica, Wetzlar, Germany).

### Apoptosis Assay

The rate of apoptosis in HaCaT cells after different treatments was evaluated using an Annexin V–FITC/propidium iodide (PI) apoptosis detection kit (BD Biosciences, 556 547). HaCaT cells (1 × 10^5^) were cultured in a six‐well plate and incubated at 37 °C for 24 h. After the specified treatment period, the cells were collected, stained using the Annexin V–FITC/PI kit, and then analyzed using flow cytometry. The data obtained were analyzed using FlowJo software.

### Luciferase Assays

Cells were transfected with p53 luciferase reporter (pP53‐TA‐luc, D2223, Beyotime) and Renilla luciferase reporter (pRL‐TK; Promega, Madison, WI, USA) and using Lipofectamine 3000 (Invitrogen, L3000‐001). Luciferase activity was measured using the Dual‐Glo Luciferase Assay System (Promega, E1910).

### TUNEL Assay

Apoptosis of paraformaldehyde‐fixed keratinocytes and skin tissue was detected using the DeadEnd colorimetric Tunel system (Promega, G3250), following the manufacturer's instructions. The detection kit utilizes the TUNEL (Terminal deoxynucleotidyl transferase dUTP Nick End Labeling) method to label fragmented DNA in apoptotic cells. The stained cells were visualized and imaged using a Nikon ECLIPSE Ni microscope.

### EdU‐Assay

Cell proliferation was detected using a cytochemical method according to the instructions provided by the manufacturer (Beyotime, C0075L). HaCaT cells were incubated with the EdU staining buffer for 2 h, followed by fixation with 4% paraformaldehyde. The cell nuclei were stained with Hoechst. Subsequently, the stained cells were examined and imaged under a microscope.

### Cell Cycle Flow Cytometry

HaCaT cells were seeded in 6‐well plates with 1.5 × 10^5^ cells per well. After 24 h, the cells were treated differently based on the experimental conditions. Cells were collected, and then re‐suspended in 500 µL of cold PBS solution. Subsequently, the cells were fixed with 70% ethanol at −20 °C for 2 days. After washing with PBS, the cells were re‐suspended in a PBS solution containing PI (BD Biosciences, 550 825) at a concentration of 20 µg mL⁻^1^ and ribonuclease A at a concentration of 10 µg mL⁻^1^. The cells were incubated in the dark for 30 min. Flow cytometry analysis was performed using a BD FACS Calibur instrument (San Jose, CA, USA), capturing at least 10 000 events. The data obtained were analyzed using FlowJo software.

### Inhibitor Experiments

Cells treated with MG132 (Selleck, S2619; 6 h, 10 µM), or Chloroquine (CQ; Selleck, S6999; 6 h, 100 nM) for the indicated times. HEK293 cells were treated with 50 µg mL⁻^1^ Cycloheximide (CHX, Selleck, S7418, 50 µg mL⁻^1^, 0, 2, 4, 6 h) for the indicated times.

### Plasmid Construction and Transfection

Flag‐tagged FGF12 was generated by PCR and then subcloned into a pcDNA3.1 eukaryotic expression vector (Invitrogen). Truncated MDM2 was amplified from vectors expressing His‐full length MDM2 and subsequently cloned into pcDNA3.1‐His expression vectors. The expression plasmids for Flag‐tagged FGF12, HA‐Ubiquitin, HA‐Ubiquitin^K48^, HA‐Ubiquitin^K63^, and His‐tagged MDM2 (His‐MDM2‐WT), along with its variants including His‐MDM2‐ΔN (1‐108aa) and His‐MDM2‐ΔR (433‐491aa), were either kindly provided or purchased from Limbio Biotechnology. The plasmids were transfected into NHEK, HEK293 and HaCat cells using jetPRIME reagent (Polyplus) according to the standard protocol.

### Immunoprecipitation

Cells were lysed using ice‐cold IP buffer (Beyotime, P10013J) with PMSF. The primary antibody was covalently immobilized on Protein A/G immunoprecipitation magnetic beads following the manufacturer's instructions (Thermo Fisher Scientific, 26 147). The cell lysates were incubated with the immobilized antibody beads for at least 4 h at 4 °C for immunoprecipitation. After immunoprecipitation, the samples were washed five times with Tris‐buffered saline. Subsequently, the immunoprecipitates were eluted with glycine‐HCl (0.1 M, pH 3.5), and the eluted proteins were subjected to immunoblotting using specific primary antibodies.

### Ubiquitination Assay

HaCaT, NHEK, and HEK293 cells were lysed with IP buffer and subsequently boiled for 10 min. For immunoprecipitation, 500 µL of the cell lysate was incubated with anti‐MDM2 or anti‐His antibody (antibody to cell lysate ratio of 1 µg mg⁻^1^) for 12 h. Afterward, 30 µL of Protein A/G immunoprecipitation magnetic beads were added and the mixture was incubated at 4 °C for 12 h. The ubiquitination of MDM2 or His‐MDM2 was then analyzed using anti‐Ub antibody western blotting.

### Protein‐Protein Docking Studies

The protein–protein docking was performed using the HDOCK server (http://hdock.phys.hust.edu.cn/). This docking program first sampled the putative binding modes between the two proteins through a fast Fourier transform (FFT) – based global search method, and then the sampled binding modes were evaluated with an improved iterative knowledge‐based scoring function for protein–protein interactions.^[^ ^51^ ^]^ PyMOL software was used to visualize the docking results of the members with the highest scores.

### RNA‐Seq Analysis and Kyoto Encyclopedia of Genes and Genomes (KEGG) Pathway Enrichment Analysis

RNA‐seq was performed at Guangzhou Epibiotek Co., Ltd., (Guangzhou, CHINA). Total RNA was isolated using Trizol reagent (Invitrogen). VAHTS Stranded mRNA‐seq Library Prep Kit for Illumina V2 (Vazyme Biotech, NR612‐02) was used for library preparation according to the instructions. Reads were aligned to the human Ensemble genome GRCh38 using Hisat2 aligner (v2.1.0) under parameters: “–rna‐strandness RF”. The reads mapped the genome were calculated using feature Counts (v1.6.3). Differential gene expression analysis was performed using the DESeq2 R‐package. KEGG pathway and Gene Ontology (GO) terms enrichment analysis of differentially expressed genes (DEGs) was carried out by cluster Profiler R Bioconductor package with a p‐value < 0.05 as statistically significant cutoffs.

### Gene Set Enrichment Analysis

Gene Set Enrichment Analysis (GSEA) was performed using the R package ClusterProfiler  (version 3.14.3), with a significance threshold set at a false discovery rate of <0.25 and P < 0.05.

### Statistical Analysis

All statistical analyses were performed using GraphPad Prism 9 software (San Diego, CA, USA) and are presented as the mean ± SEM. The results were analyzed using two‐tailed, unpaired Student's t test (two groups) or one‐way ANOVA (more than two groups). A *P* value of <0.05 was considered to indicate statistical significance.

## Conflict of Interest

The authors declare no conflict of interest.

## Author Contributions

N.W., Z.Z., W.C., and X.L., designed the research and obtained material support and study supervision. N.W., X.X., and F.G., performed the cell and animal studies. N.W., Y.L., Y.Y., J.Z., J.F., S.L., J.Y., and Z.T., performed the fluorescence experiments. N.W., W.G., B.S., Y.S., L.S., Y.S., and L.J., analyzed the data, wrote and edited the manuscript. All authors reviewed the manuscript.

## Supporting information

Supporting Information

## Data Availability

The data that support the findings of this study are available on request from the corresponding author. The data are not publicly available due to privacy or ethical restrictions.
