# Supplementary material for: FGF12 Positively Regulates Keratinocyte Proliferation by Stabilizing MDM2 and Inhibiting p53 Activity in Psoriasis
Source: Adv Sci (Weinh). 2024 Sep 5;11(39):2400107. doi: 10.1002/advs.202400107 (PMC11497104; doi:10.1002/advs.202400107)
Supplement: Supplementary file 1 — Supporting Information [file ADVS-11-2400107-s001.docx]

Supporting Information

**FGF12 positively regulates keratinocyte proliferation by stabilizing MDM2 and inhibiting p53 activity in psoriasis**

***Authors***

*Nan Wang,* ***Xiejun Xu, Fangqian Guan, Yifan Lin, Yizhou Ye, Jie Zhou, Jianjun Feng, Sihang Li, Junbo Ye, Zhouhao Tang, Wenjie Gao, Bohao Sun, Yingjie Shen, Li Sun, Yonghuan Song, Litai Jin, Xiaokun Li^*^, Weitao Cong^*^, Zhongxin Zhu^*^.***

**AFFILIATIONS**

**N. Wang, X. Xu, F. Guan, Y. Lin, Y. Ye, J. Zhou, J. Feng, S. Li, J. Ye, W. Gao, L. Jin, X. Li, W. Cong, Z. Zhu**

**School of Pharmaceutical Science, Wenzhou Medical University, Wenzhou 325035, China**

**E-mail:Xiaokunli@wmu.edu.cn;cwt97126@wmu.edu.cn; zhongxinzhu@wmu.edu.cn.**

**N. Wang**

**Department of Pharmacy, Zhejiang Provincial People’s Hospital (Affiliated People’s Hospital, Hangzhou Medical College), Hangzhou 310014, China**

1. **Tang**

**Department of Cardiology, The Second Affiliated Hospital and Yuying Children's Hospital of Wenzhou Medical University, Wenzhou 325027, China**

1. **Sun**

**Department of Pathology, The Second Affiliated Hospital of Zhejiang University, Hangzhou 310009, China**

1. **Shen**

**School of Life Sciences, Huzhou University, Huzhou 313000, China**

1. **Sun**

**Department of Rheumatology and Immunology, The First Affiliated Hospital of Wenzhou Medical University, Wenzhou 325000, China**

**Y. Song**

**Department of Orthopaedics, The Second Affiliated Hospital and Yuying Children's Hospital of Wenzhou Medical University, Wenzhou 325027, China**

**This file includes the following subsections:**

**Supplementary materials;**

**Supplementary Figures S1-S10;**

**Supplementary Table S1;**

**Supplementary Table S2**

**SUPPLEMENTARY FIGURES**


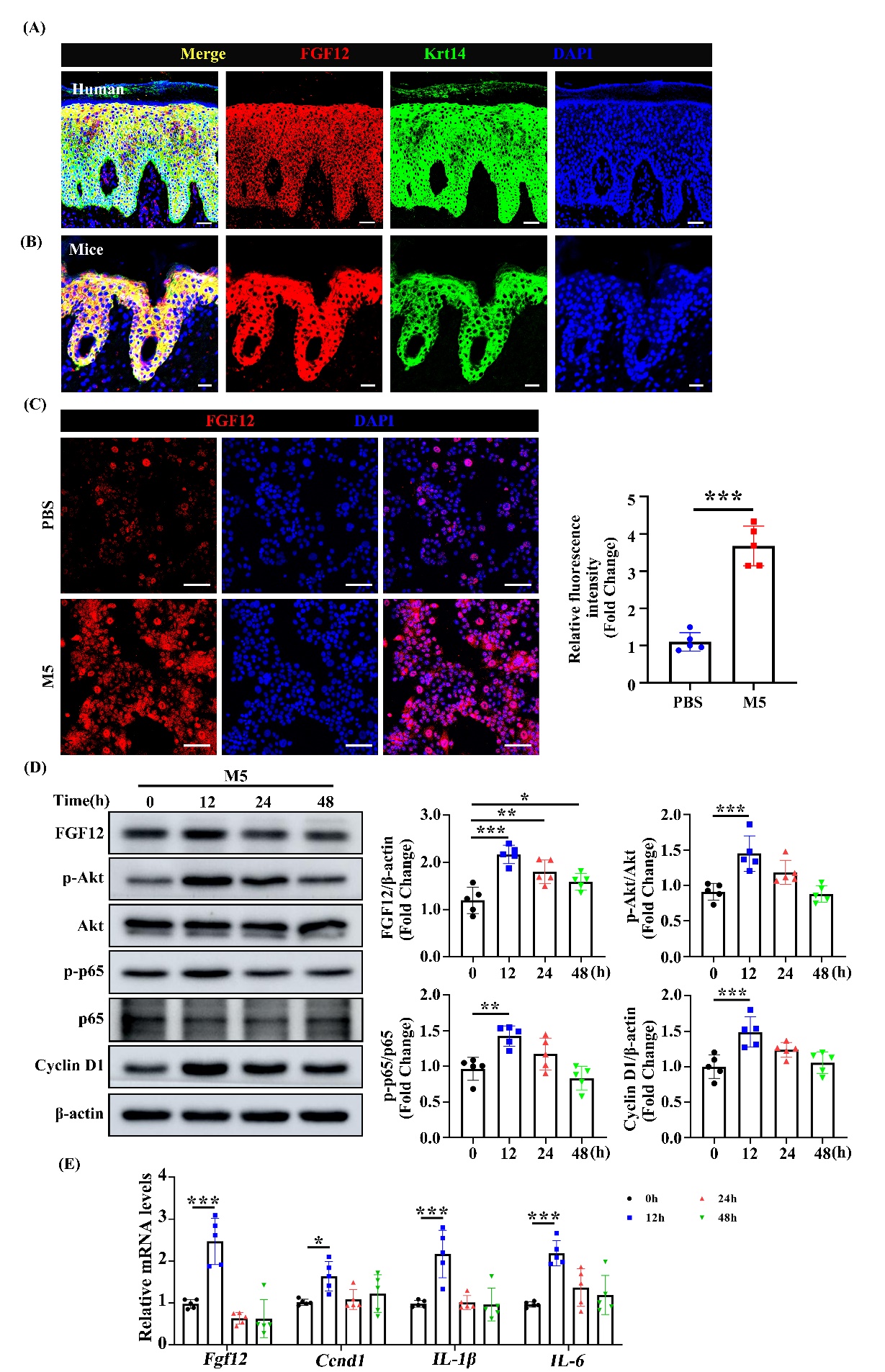


**Supplementary Figure S1: FGF12 is upregulated in the epidermis of patients with psoriasis and the IMQ-induced mouse model.**

**(A)** Immunofluorescence images of human skin was labeled with FGF12 antibody. Sections were co-stained with DAPI to visualize nuclei and Krt14 to visualize keratinocytes. Scar bar = 50 μm. **(B)** Immunofluorescence images of mice skin was labeled with FGF12 antibody. Sections were co-stained with DAPI to visualize nuclei and Krt14 to visualize keratinocytes. Scar bar = 20 μm. **(C)** Immunofluorescent and quantitative analysis of FGF12 in HaCaT cells that treated with PBS or M5 for 12 h. Nuclei were stained with DAPI (blue) (n = 5). Scale bar = 100 µm. **(D)** Immunoblotting and quantitative analysis of FGF12, p-Akt, Akt, p-p65, p65 and Cyclin D1 protein levels in HaCaT cells stimulates with M5 for 0, 12, 24 and 48 h treatment. β-Actin was used as a loading control (n = 5). **(E)** qRT-PCR analysis for *Fgf12*, *Ccnd1*, *IL-1β* and *IL-6* mRNA levels in HaCaT cells stimulates with M5 for 0, 12, 24 and 48 h treatment (n = 5). Error bars show the mean ± SEM. ** P* < 0.05; *** P* < 0.01; **** P* < 0.001. The *P* value was determined using two-tailed Student’s t test **(C)** or one-way ANOVA **(D** and **E)**. All numbers (n) are biologically independent experiments.

**
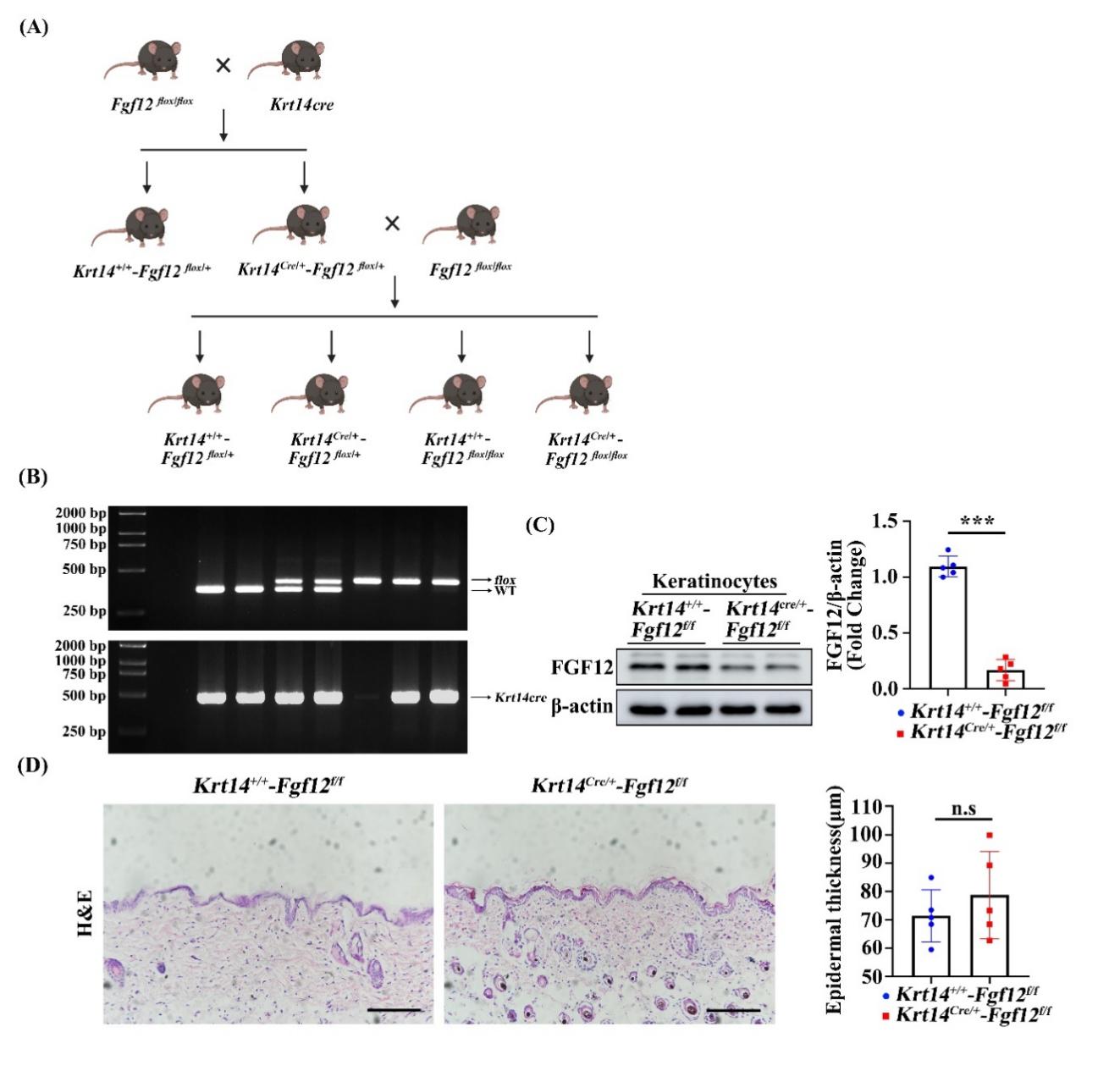
**

**Supplementary Figure S2: Targeted disruption of the *Fgf12* gene in keratinocytes.**

**(A)** Illustration of the mouse crossbreeding strategy. **(B)** Representative PCR genotyping of *Fgf12 flox/flox* mice and *Krt14 Cre* mice. **(C)** Immunoblotting analysis of FGF12 expression in keratinocytes derived from control and keratinocytes-specific FGF12 knockout mice. β-Actin was used as a loading control (n = 5). **(D)** Representative histological sections of the dorsal back from *Krt14^+/+^-Fgf12^f/f^* and *Krt14^Cre/+^-Fgf12^f/f^* mice stained with H&E, and quantification of the epidermal thickness stained with H&E (n = 5). Scale bars = 100 μm. Error bars show the mean ± SEM. **** P* < 0.001; n.s., not significant. The *P* value was determined using two-tailed unpaired Student’s t test **(C** and **D)**. All numbers (n) are biologically independent experiments.


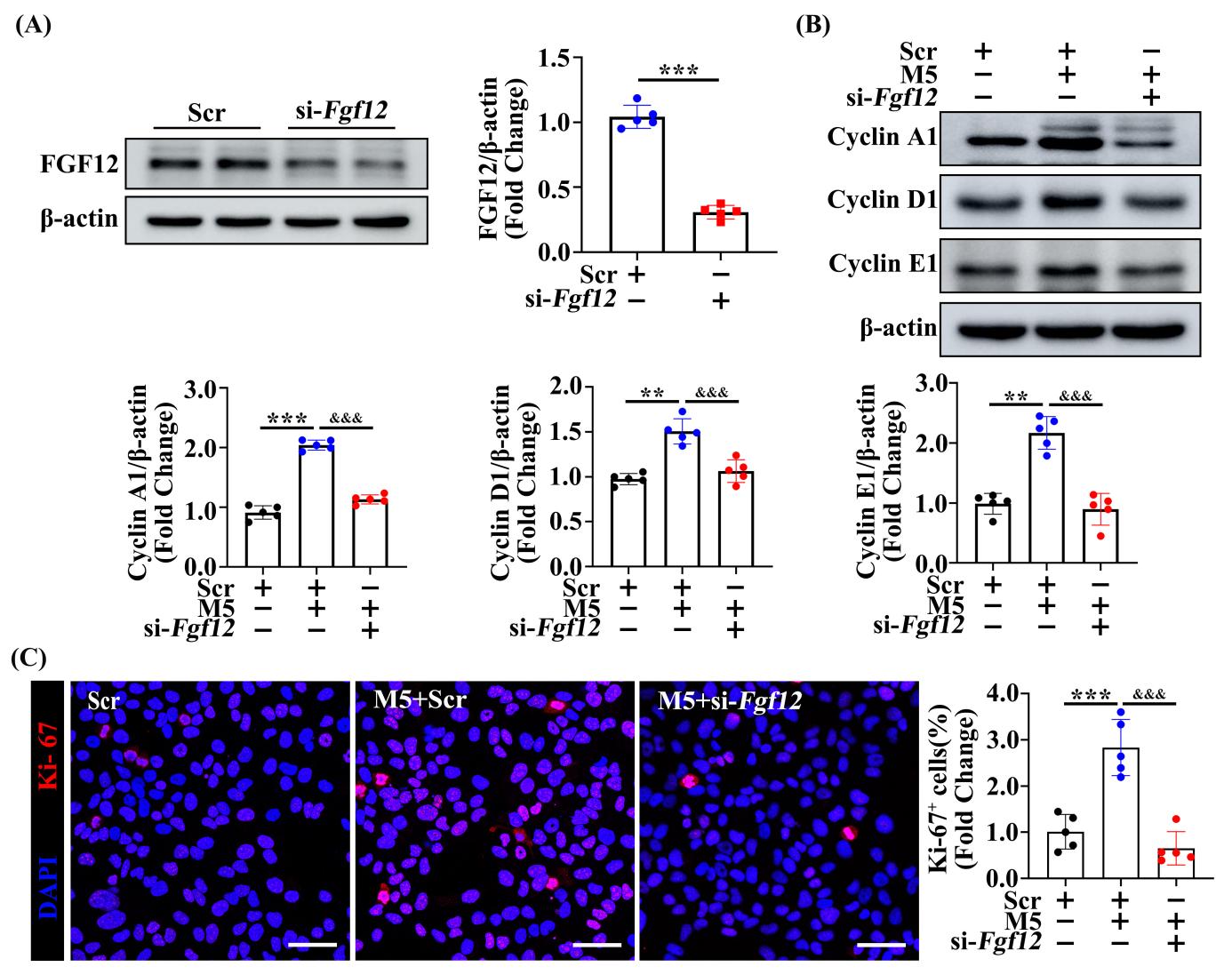


**Supplementary Figure S3: Knockdown of FGF12 in HaCaT cells was shown to reduce keratinocyte proliferation in psoriasis.**

1. Immunoblotting and quantitative analysis of FGF12 protein level in HaCaT cells that were transfected in si-Scr (Scramble) or si-*Fgf12*. β-Actin was used as a loading control (n = 5). **(B)** Immunoblotting and quantitative analysis of Cyclin A1, Cyclin D1, and Cyclin E1 protein levels in HaCaT cells that were treated with si-Scr or si-*Fgf12* and stimulated with or without M5 for 12 h. β-Actin was used as a loading control (n = 5). **(C)** Immunofluorescent and quantitative analysis of Ki-67^+^ in HaCaT cells that were treated with si-Scr or si-*Fgf12* and stimulated with or without M5 for 12 h. Nuclei were stained with DAPI (blue) (n = 5). Scale bar = 100 µm. Error bars show the mean ± SEM. *** P* < 0.01; **** P* < 0.001. *^&&&^ P* < 0.001. The *P* value was determined using two-tailed unpaired Student’s t test **(A)** or one-way ANOVA **(B** and **C)**. All numbers (n) are biologically independent experiments.


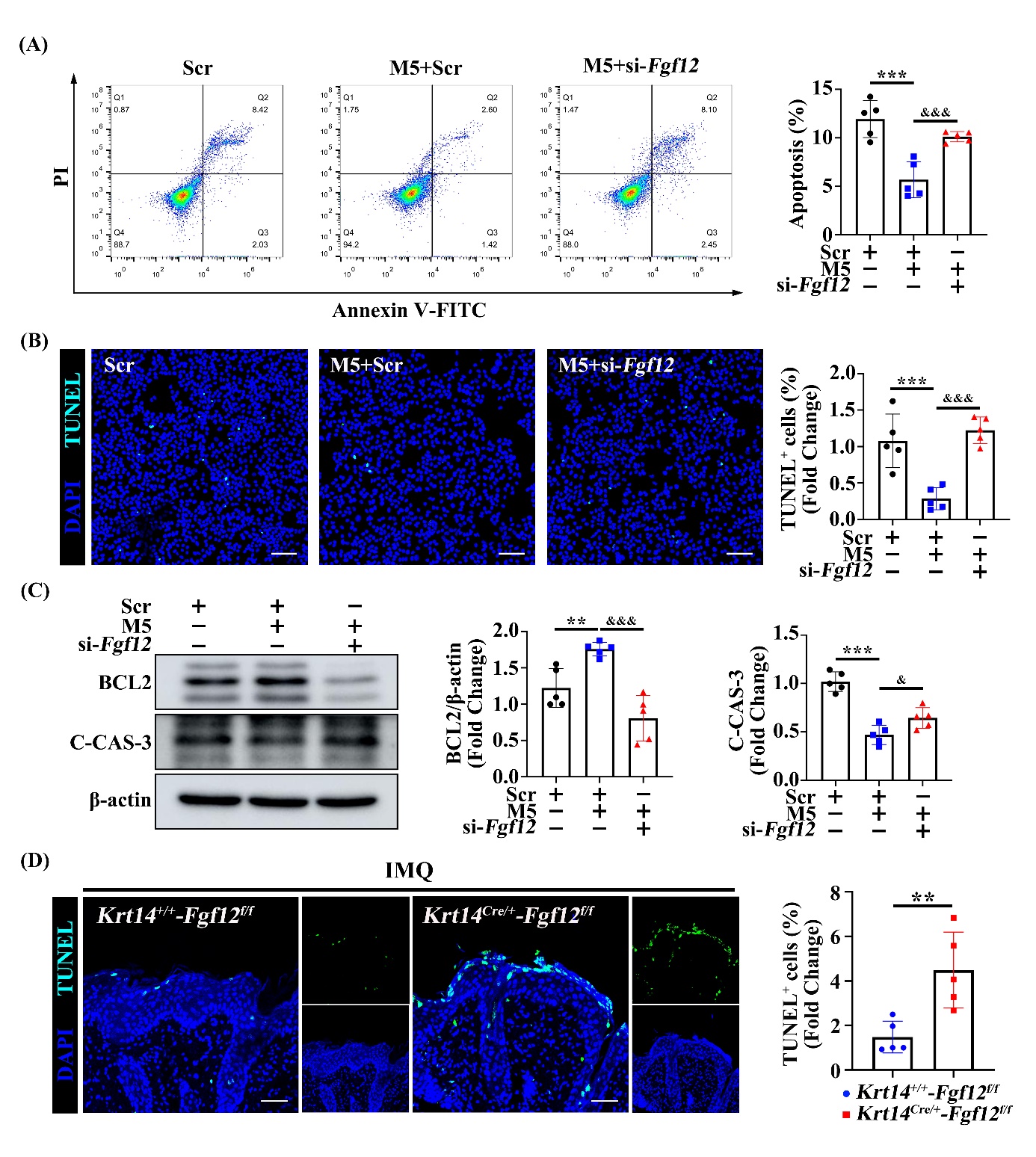


**Supplementary Figure S4: FGF12 depletion in keratinocytes upregulates apoptosis.**

1. Apoptotic cells were monitored by Annexin V-FITC/PI staining and flow-cytometry analysis that HaCaT cells treated with si-Scr or si-*Fgf12* and stimulated with or without M5 for 12 h (n = 5). **(B)** TUNEL staining was performed on HaCaT cells treated with si-Scr or si-*Fgf12* and stimulated with or without M5 for 12 h. The TUNEL^+^ nuclei (green) indicated the apoptotic cells. DAPI (blue) indicated all cell nuclei (n = 5). Scale bar = 100 μm. **(C)** Immunoblotting and quantitative analysis of BCL2, and C-CAS-3 protein levels in HaCaT cells that were treated with si-Scr or si-*Fgf12* and stimulated with or without M5 for 12 h. β-Actin was used as a loading control (n = 5). **(D)** TUNEL staining was performed on the dorsal back from *Krt14^+/+^-Fgf12^f/f^* and *Krt14^Cre/+^-Fgf12^f/f^* mice treated by IMQ. The TUNEL^+^ nuclei (green) indicated the apoptotic cells. DAPI (blue) indicated all cell nuclei (n = 5). Scale bar = 50 μm. Error bars show the mean ± SEM. *** P* < 0.01; **** P* < 0.001. *^&^ P* < 0.05; *^&&&^ P* < 0.001. The *P* value was determined using two-tailed unpaired Student’s t test **(D)** or one-way ANOVA **(A-C)**. All numbers (n) are biologically independent experiments.


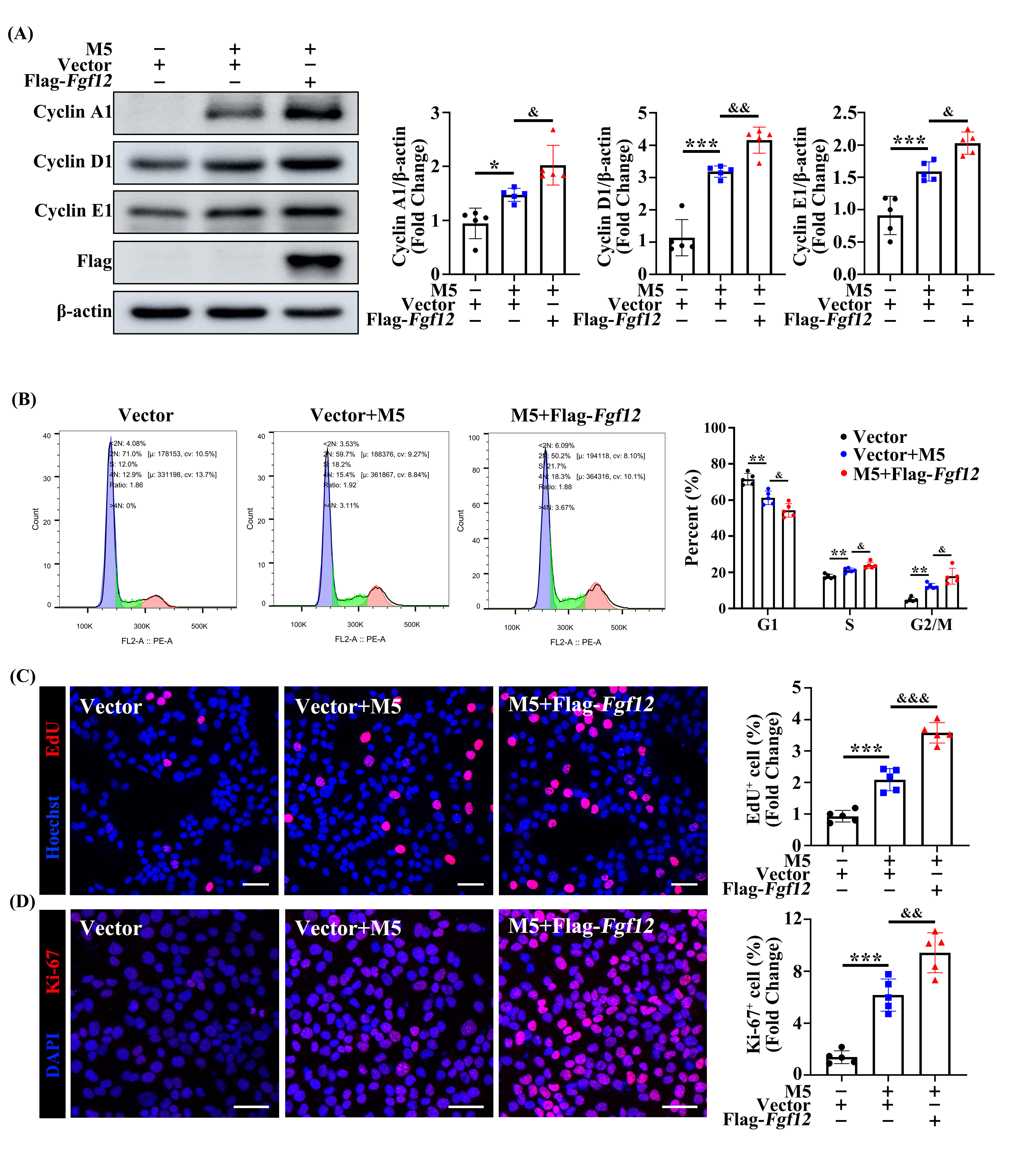


**Supplementary Figure S5: Overexpression of FGF12 in keratinocytes enhances proliferation and worsens the symptoms of psoriasis.**

1. Immunoblotting and quantitative analysis of Cyclin A1, Cyclin D1, and Cyclin E1 protein levels in HaCaT cells that were transfected in Vector or Flag-*Fgf12* plasmid and stimulated with or without M5 for 12 h. β-Actin was used as a loading control (n = 5). **(B)** Flow cytometric plots of cell-cycle analysis performed with PI staining on HaCaT cells that were transfected in Vector or Flag-*Fgf12* plasmid and stimulated with or without M5 for 12 h (n = 5). Quantification the percentage of cells that fall into the sub G0/G1, S, or G2/M gates (right). **(C)** Immunofluorescent and quantitative analysis of EdU^+^ in HaCaT cells that transfected in Vector or Flag-*Fgf12* plasmid and stimulated with or without M5 for 12 h. Nuclei were stained with Hoechst (blue) (n = 5). Scale bar = 50 µm. **(D)** Immunofluorescent and quantitative analysis of Ki-67^+^ in HaCaT cells that transfected in Vector or Flag-*Fgf12* plasmid and stimulated with or without M5 for 12 h. Nuclei were stained with DAPI (blue) (n = 5). Scale bar = 50 µm. Error bars show the mean ± SEM. ** P* < 0.05, *** P* < 0.01; **** P* < 0.001. *^&^ P* < 0.05; *^&&^ P* < 0.01; *^&&&^ P* < 0.001. The *P* value was determined using one-way ANOVA **(A-D)**. All numbers (n) are biologically independent experiments.

**
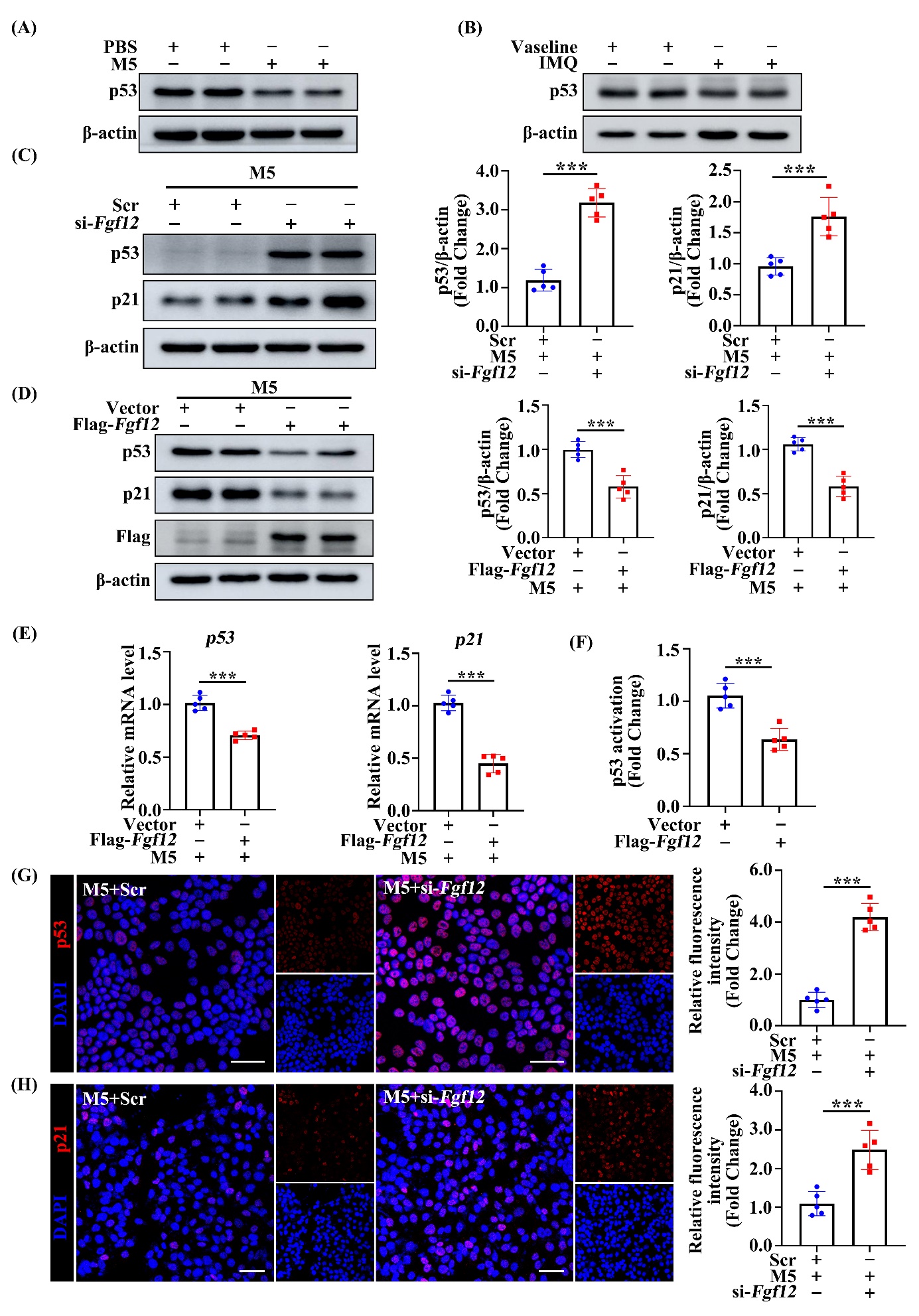
Supplementary Figure S6: FGF12 mainly regulates the p53 signaling pathway in psoriatic** **keratinocytes.**

1. Immunoblotting analysis of p53 protein level in HaCaT cells that was stimulated with PBS or M5 for 12 h. β-Actin was used as a loading control. **(B)** Immunoblotting analysis of p53 protein level in Vaseline and IMQ-treated mice. β-Actin was used as a loading control. **(C)** Immunoblotting and quantitative analysis of p53 and p21 protein levels in HaCaT cells that were treated with si-Scr or si-*Fgf12*. β-Actin was used as a loading control (n = 5). **(D)** Immunoblotting and quantitative analysis of p53 and p21 protein levels in HaCaT cells that were transfected in Vector or Flag-*Fgf12* plasmid and stimulated with M5 for 12 h. β-Actin was used as a loading control (n = 5). **(E)** qRT-PCR analysis for *p53* and *p21* mRNA levels in HaCaT cells that were transfected in Vector or Flag-*Fgf12* plasmid and stimulated with M5 for 12 h (n = 5). **(F)** p53-dependent transcriptional activity of p53 determined by performing dual-luciferase assays with HEK293 cells overexpressing FGF12 (n = 5). **(G)** Immunofluorescent and quantitative analysis of p53 in HaCaT cells that transfected in si-Scr or si-*Fgf12* and stimulated with M5 for 12 h. Nuclei were stained with DAPI (blue) (n = 5). Scale bar = 50 µm. **(H)** Immunofluorescent and quantitative analysis of p21 in HaCaT cells that transfected in si-Scr or si-*Fgf12* and stimulated with M5 for 12 h. Nuclei were stained with DAPI (blue) (n = 5). Scale bar = 50 µm. Error bars show the mean ± SEM. **** P* < 0.001. The *P* value was determined using two-tailed unpaired Student’s t test **(C-H)**. All numbers (n) are biologically independent experiments.

**
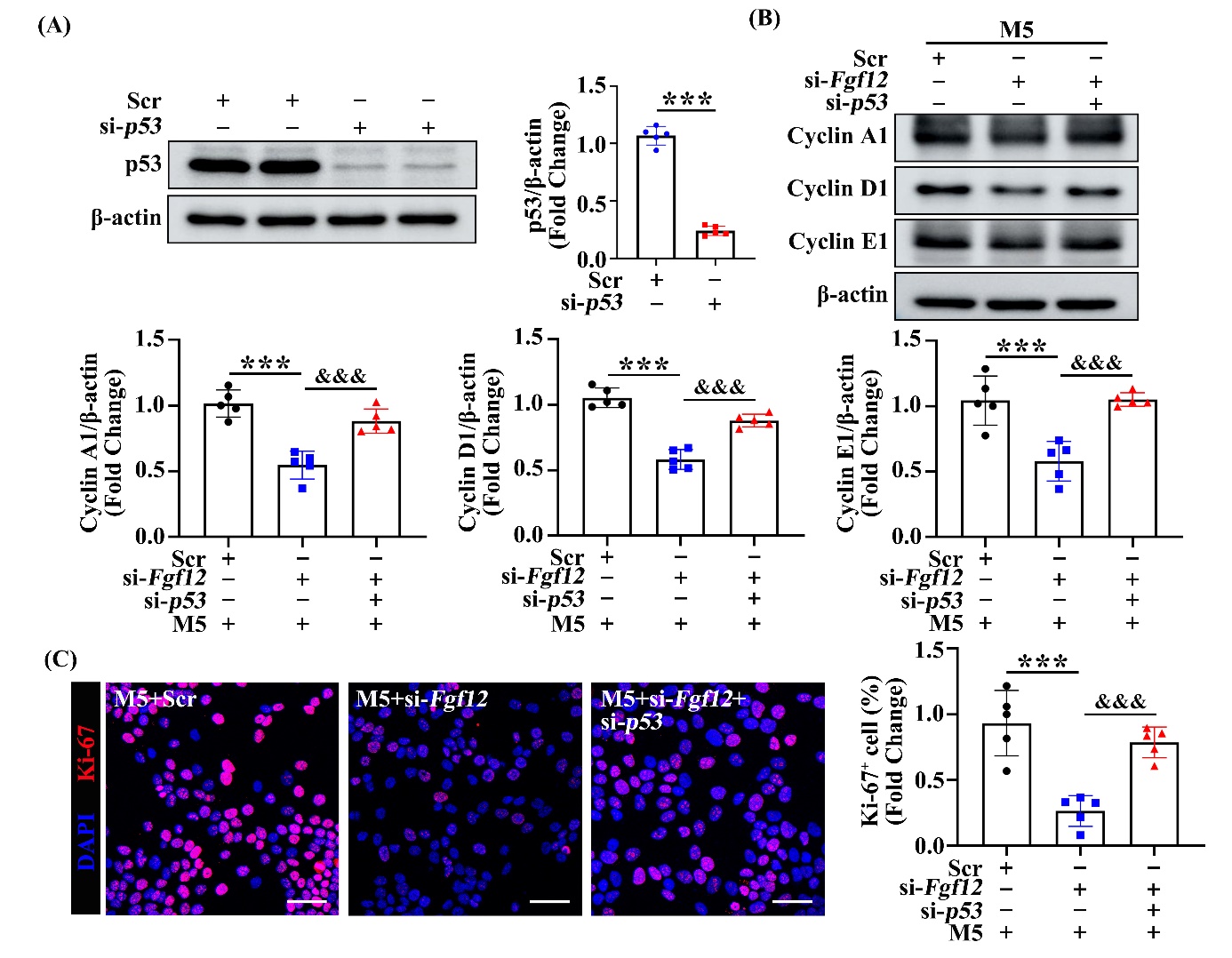
**

**Supplementary Figure S7: The role of FGF12-dependent p53 in promoting keratinocyte proliferation in psoriasis was confirmed in HaCaT cells.**

1. Immunoblotting and quantitative analysis of p53 protein level in HaCaT cells that were treated with si-Scr or si-*p53*. β-Actin was used as a loading control (n = 5). **(B)** Immunoblotting and quantitative analysis of Cyclin A1, Cyclin D1, and Cyclin E1 protein levels in HaCaT cells that si-*p53* or si-Scr was transfected in FGF12-interference cells treated with M5 for 12 h. β-Actin was used as a loading control (n = 5). **(C)** Immunofluorescent and quantitative analysis of Ki-67^+^ in HaCaT cells that si-*p53* or si-Scr was transfected in FGF12-interference cells treated with M5 for 12 h. Nuclei were stained with DAPI (blue) (n = 5). Scale bar = 50 µm. Error bars show the mean ± SEM. **** P* < 0.001. *^&&&^ P* < 0.001. The *P* value was determined using two-tailed unpaired Student’s t test **(A)** or one-way ANOVA **(B** and **C)**. All numbers (n) are biologically independent experiments.


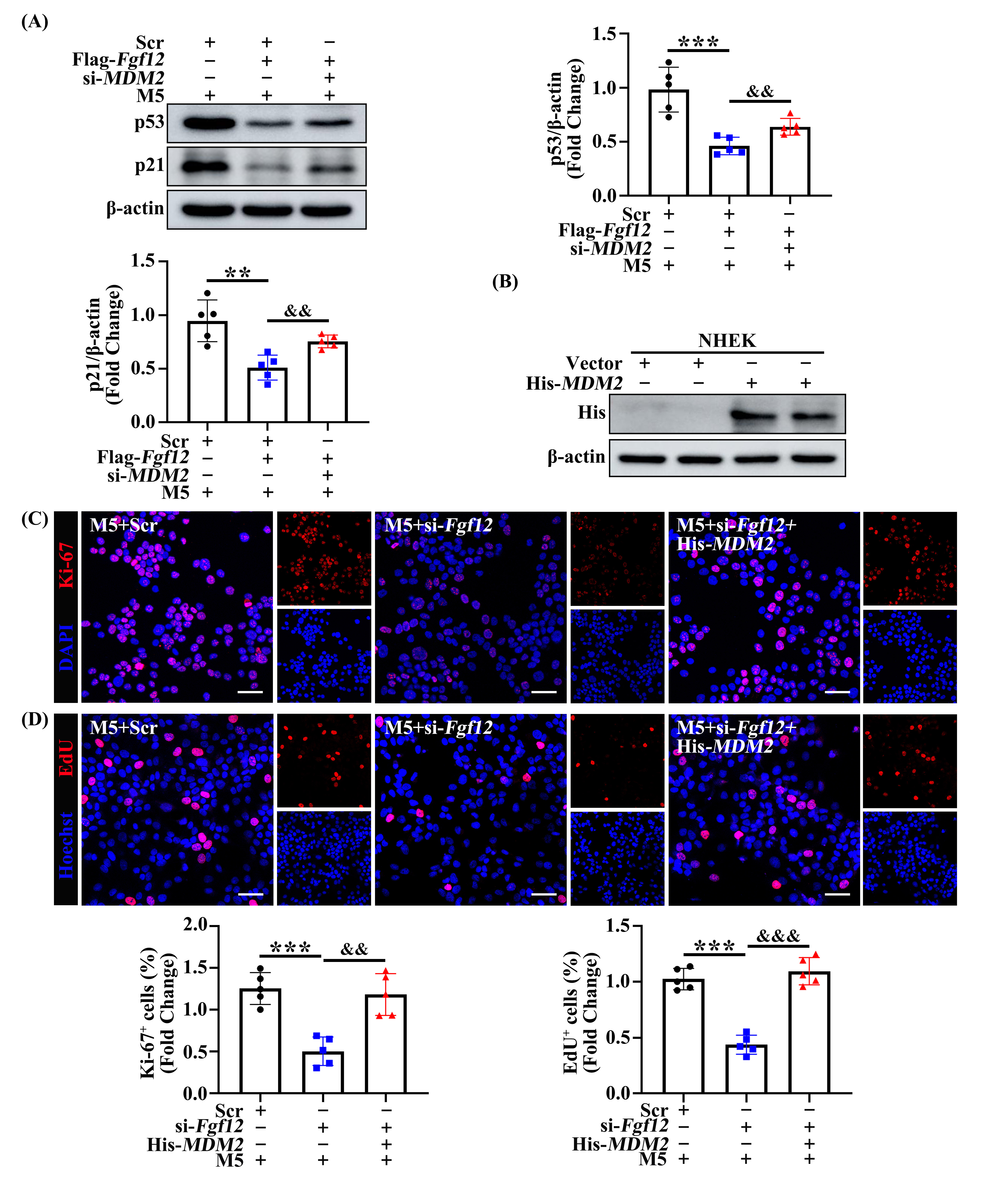


**Supplementary Figure S8: MDM2 is involved in the regulation of keratinocyte proliferation by FGF12.**

1. Immunoblotting and quantitative analysis of p53 and p21 protein levels in HaCaT cells that si-*MDM2* or si-Scr was transfected in Flag-*Fgf12* cells treated with M5. β-Actin was used as a loading control (n = 5). **(B)** Immunoblotting and quantitative analysis of His protein level in NHEK cells that were transfected in Vector or His-*MDM2*. β-Actin was used as a loading control. **(C)** Immunofluorescent and quantitative analysis (bottom) of Ki-67^+^ in HaCaT cells that His-*MDM2* or Vector was transfected in si-*Fgf12* cells treated with M5 for 12 h. Nuclei were stained with DAPI (blue) (n = 5). Scale bar = 50 µm. **(D)** Immunofluorescent and quantitative analysis (bottom) of EdU^+^ in HaCaT cells that His-*MDM2* or Vector was transfected in si-*Fgf12* cells treated with M5 for 12 h. Nuclei were stained with Hoechst (blue) (n = 5). Scale bar = 50 µm. Error bars show the mean ± SEM. *** P* < 0.01; **** P* < 0.001. *^&&^ P* < 0.01; *^&&&^ P* < 0.001. The *P* value was determined using one-way ANOVA **(A, C** and **D)**. All numbers (n) are biologically independent experiments.


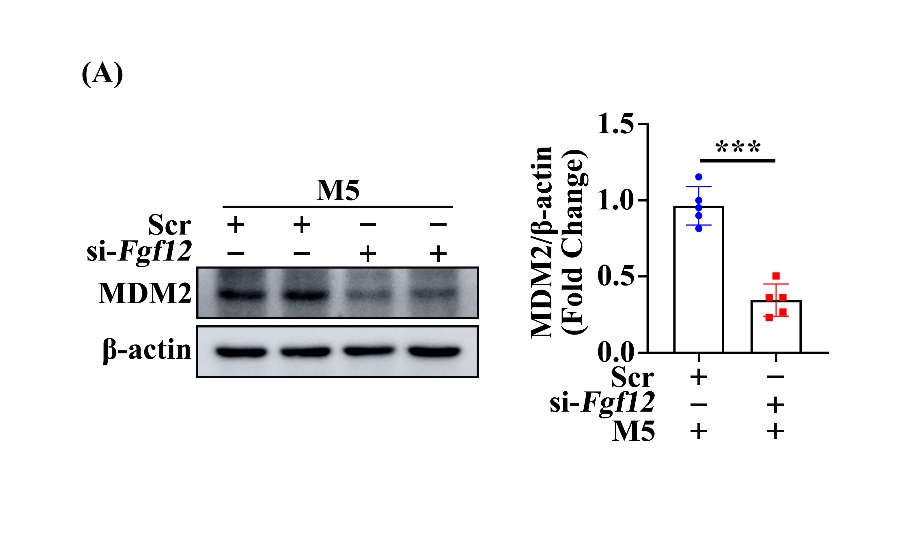


**Supplementary Figure S9: FGF12 positively regulates MDM2 protein expression in psoriatic keratinocytes.**

1. Immunoblotting and quantitative analysis of MDM2 protein level in HaCaT cells that were transfected in si-Scr or si-*Fgf12* and stimulated with M5 for 12 h. β-Actin was used as a loading control (n = 5). Error bars show the mean ± SEM. **** P* < 0.001. The *P* value was determined using two-tailed unpaired Student’s t test **(A)**. All numbers (n) are biologically independent experiments.

**
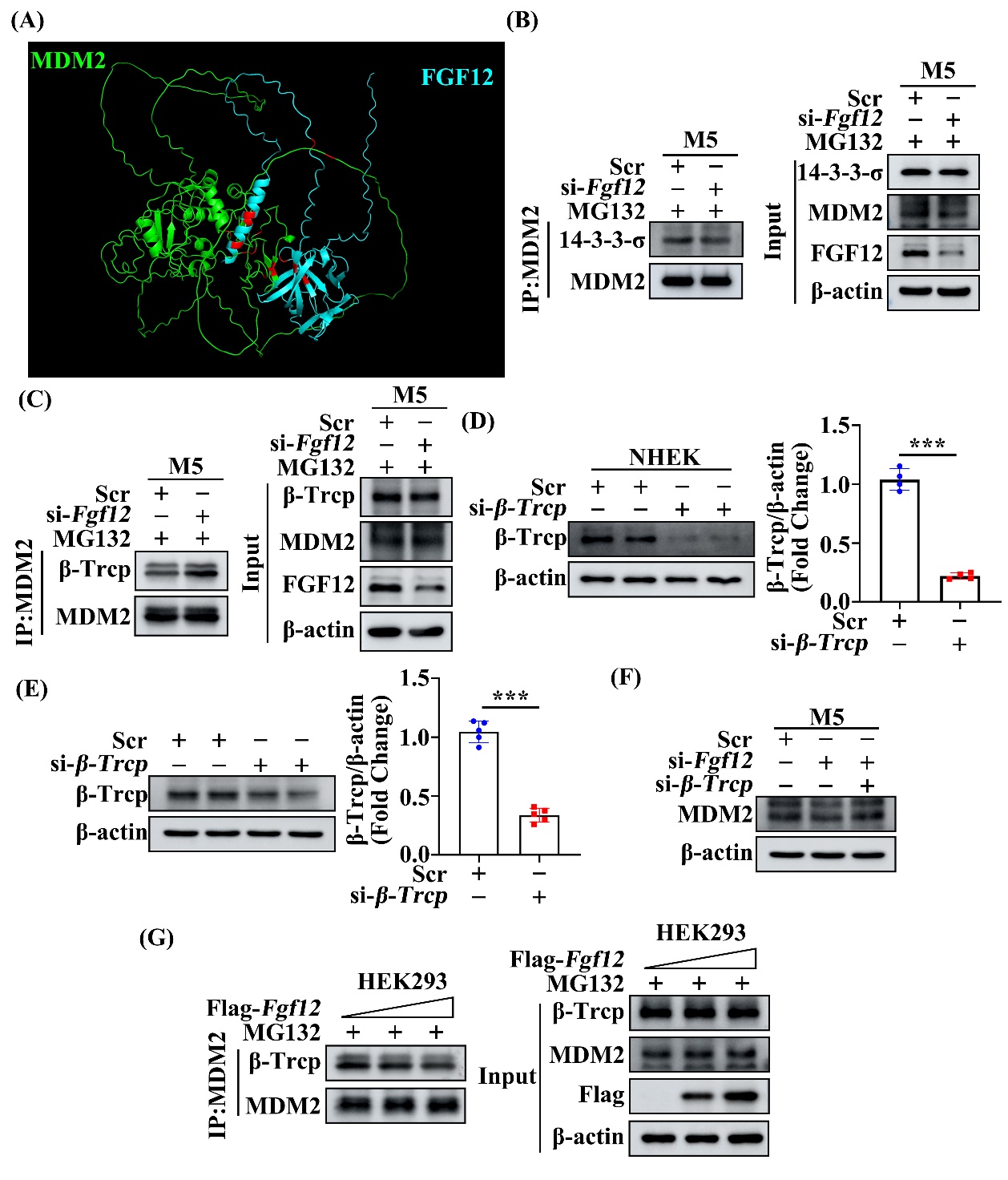
**

**Supplementary Figure S10: FGF12 interacts with MDM2 and thereby blocks binding of β-Trcp.**

1. The protein-protein docking was performed using the HDOCK server. Model showing the probable interaction of MDM2 (green ribbon) with FGF12 (blue ribbon) and binding site (red region). (Interaction confidence analysis: MDM2 - FGF12: 0.9306). **(B)** HaCaT cells were transfected with si-Scr or si-*Fgf12* and stimulated with M5 for 12 h. Cells were treated with MG132 (10 μM) for 6 h before lysation. Whole-cell lysates were IP with anti-MDM2 then subjected to immunoblot analysis with the anti-14-3-3-σ, anti-FGF12 and anti-MDM2 antibodies. **(C)** HaCaT cells were transfected with si-Scr or si-*Fgf12* and then stimulated with M5 for 12 h. Cells were treated with MG132 (10 μM) for 6 h before lysation. Whole-cell lysates were immunoprecipitated with anti-MDM2 then subjected to immunoblot analysis with the anti-β-Trcp, anti-MDM2 and anti-FGF12 antibodies. **(D)** Immunoblotting and quantitative analysis of β-Trcp protein level in NHEK cells that were transfected with si-Scr and si-*β-Trcp*. β-Actin was used as a loading control (n = 4). **(E)** Immunoblotting and quantitative analysis of β-Trcp protein level in HaCaT cells that were transfected with si-Scr or si-*β-Trcp*. β-Actin was used as a loading control (n = 5). **(F)** Immunoblotting analysis of MDM2 protein level in HaCaT cells that were transfected with si-Scr, si-*β-Trcp* and si-*Fgf12* treatment by M5 for 12 h. β-Actin was used as a loading control. **(G)** HEK293 cells were co-transfected with Flag-*Fgf12* plasmid (0, 2, 4 μg) for 48 h, and then the cells were treated with MG132 (10 μM) for 6 h. Whole-cell lysates were IP with anti-MDM2 then subjected to immunoblotting analysis with the anti-β-Trcp, anti-MDM2 and anti-Flag antibodies. Error bars show the mean ± SEM. **** P* < 0.001. The *P* value was determined using two-tailed unpaired Student’s t test **(D** and **F)**. All numbers (n) are biologically independent experiments.

**Supplementary Table-S1. List of antibodies used in the western blot.**

| **Antibody** | **Manufacture** | **Cat No.** | **Dilution** |
| --- | --- | --- | --- |
| FGF12  p-Akt  Akt  p-p65  p65  Cyclin A1  Cyclin D1  Cyclin E1  β-actin  p53  p21  MDM2  His-tag  HA-tag  Ubiquitin  Flag-tag  BCL-2  **C-CAS-3**  β-Trcp  14-3-3-σ | Abcam  Cell Signaling Technology  Cell Signaling Technology  Cell Signaling Technology  Cell Signaling Technology  Cell Signaling Technology  Abcam  Cell Signaling Technology  Cell Signaling Technology  Cell Signaling Technology  Cell Signaling Technology  Abcam  Abcam  Abcam  Abcam  Sigma  Cell Signaling Technology  Cell Signaling Technology  SantaCruz Biotechnology  SantaCruz Biotechnology | ab231956  4060  4691  3033  8242  17416  ab16663  20808  3700  2524  2947  ab259265  ab18184  ab9110  ab134953  F1804  3498  9664  sc-390629  sc-166473 | 1:1000  1:1000  1:1000  1:1000  1:1000  1:1000  1:200  1:1000  1:1000  1:1000  1:1000  1:1000  1:2000  1:5000  1:5000  1:5000  1:1000  1:1000  1:1000  1:1000 |

Abbreviations: FGF12, Fibroblast growth factor 12; MDM2, mouse double minute 2;

C-CAS-3, Cleaved Caspase-3.

**Supplementary Table-S2. Primer sequences used for reverse transcription-quantitative PCR.**

| **Gene Name** | **Forward Primer** | **Reverse Primer** |
| --- | --- | --- |
| ***Fgf12 (h)*** | GGGACCAAGGACGAAAACAG | TTGCTGGCGGTACAGTGTG |
| ***MDM2 (h)*** | CAGTAGCAGTGAATCTACAGGGA | CTGATCCAACCAATCACCTGAAT |
| ***p53 (h)*** | CAGCACATGACGGAGGTTGT | TCATCCAAATACTCCACACGC |
| ***p21 (h)*** | TGTCCGTCAGAACCCATGC | AAAGTCGAAGTTCCATCGCTC |
| ***β-actin (h)*** | CCACGAAACTACCTTCAACTCC | GTGATCTCCTTCTGCATCCTGT |
| ***Ccnd1 (h)*** | ATGCCAACCTCCTCAACGAC | GGACCTCCTTCTGCACACAT |
| ***Il-1β (h)*** | CTTCAGCCAATCTTCATT | ATTGCCACTGTAATAAGC |
| ***Il-6 (h)*** | ACTCACCTCTTCAGAACGAATTG | CCATCTTTGGAAGGTTCAGGTTG |
| ***IL-17 (m)*** | TACCTCAACCGTTCCACGTC | TTTCCCTCCGCATTGACACA |
| ***CCL20 (m)*** | AATCTGTGTGCGCTGATCCA | CCTTGGGCTGTGTCCAATTC |
| ***S100A8 (m)*** | AAGACATCGTTTGAAAGGAAA | TGGTAGACATCAATGAGGTT |
| ***CXCL2 (m)*** | CAGACAGAAGTCATAGCCAC | TTCCAGGTCAGTTAGCCTTG |
| ***IL-1β (m)*** | TGCCACCTTTTGACAGTGATG | TGATGTGCTGCTGCGAGATT |
| ***Fgf12 (m)*** | CTCGGGGTGTTCAGCAAAGT | TTTCGTCCTTGGTCCCATCAA |
| ***β-actin (m)*** | CCTCTATGCCAACACAGTGC | ACATCTGCTGGAAGGTGGAC |
| ***Fgf12 flox*** | P1 | GGGCTCAAACCCAACAAGTG |
|  | P2 | GGGAGGCAGGGAGAGAAAGAA |
| ***Krt14-Cre*** | P1 | TTCCTCAGGAGTGTCTTCGC |
|  | P2 | GTCCATGTCCTTCCTGAAGC |
